# Development of scalable and generalizable machine learned force field for polymers

**DOI:** 10.1038/s41598-023-43804-5

**Published:** 2023-10-11

**Authors:** Shaswat Mohanty, James Stevenson, Andrea R. Browning, Leif Jacobson, Karl Leswing, Mathew D. Halls, Mohammad Atif Faiz Afzal

**Affiliations:** 1 Schrödinger, Inc., Portland, OR 97204 USA; 2 Schrödinger, Inc., New York, 10036 USA; 3 Schrödinger, Inc., San Diego, CA 92121 USA

**Keywords:** Condensed-matter physics, Soft materials, Theory and computation, Computational science

## Abstract

Understanding and predicting the properties of polymers is vital to developing tailored polymer molecules for desired applications. Classical force fields may fail to capture key properties, for example, the transport properties of certain polymer systems such as polyethylene glycol. As a solution, we present an alternative potential energy surface, a charge recursive neural network (QRNN) model trained on DFT calculations made on smaller atomic clusters that generalizes well to oligomers comprising larger atomic clusters or longer chains. We demonstrate the validity of the polymer QRNN workflow by modeling the oligomers of ethylene glycol. We apply two rounds of active learning (addition of new training clusters based on current model performance) and implement a novel model training approach that uses partial charges from a semi-empirical method. Our developed QRNN model for polymers produces stable molecular dynamics (MD) simulation trajectory and captures the dynamics of polymer chains as indicated by the striking agreement with experimental values. Our model allows working on much larger systems than allowed by DFT simulations, at the same time providing a more accurate force field than classical force fields which provides a promising avenue for large-scale molecular simulations of polymeric systems.

## Introduction

Machine learning techniques to bridge computational shortcomings have been explored in numerous fields of computational materials science^1–4^ and chemistry^5–9^. In particular, there has been a recent foray into the field of molecular dynamics (MD) to (i) lengthen the time or length scale associated with ab-initio molecular dynamics, and (ii) to improve the accuracy of classical force fields. In the current work, we will primarily focus on the properties with limited accuracy that are associated with classical force fields. To address this issue, *ab initio* molecular dynamics (AIMD) methods were developed^10, 11^, primarily using density functional theory (DFT)^12^. While these methods accurately capture molecular interactions, the computational expense associated with their scalability restricts us to short time scales (around $10^1$ picoseconds) and very small systems (around $10^2$ atoms), which complicates the study of time-dependent dynamic properties of systems of large molecules such as polymers^13–15^.

There have been recent advancements in machine-learned force fields^16, 17^ to capture complex behavior such as polarization and chemical reactivity^18^. Machine-learned force fields can reproduce quantum chemical calculations in both finite and extended systems to well within chemical accuracy, i.e., less than 2 kcal/mol error. These force fields are based on a neural network potential energy surface (NN-PES) architecture, in which a machine learning model is trained to reproduce the total electronic energy of a reference level of theory (typically DFT) to chemical accuracy^19–21^. The model is trained by minimizing the prediction error against the reference level of theory. In the approach known as a high dimensional neural network potential (HDNNP)^22, 23^, this is done by first transforming the atomic positions into local atomic descriptors which can then be mapped to atomic energy contributions. The total energy is then computed as the sum of the atomic energies. Typically HDNNPs are applied to the same chemical systems that they are trained on, enabling simulations at larger length scales and timescales which would not have been possible using traditional *ab initio* molecular dynamics techniques^24^. Recent efforts have been made to further improve HDNNP architecture and also extend to systems that are not part of the training set^25^. Most notably, the charge recursive neural network (QRNN) model^26^ aims to supplement and build on the success of Accurate NeurAl networK engINe for Molecular Energies (ANI)^16^ by distinguishing charged species from uncharged species while keeping the generalizability that allows accuracy without task-specific training. By generalizing from clusters of small molecules up to large periodic boxes, we can use an advanced hybrid density functional like $\omega$B97X-D3BJ^27, 28^ to generate training data, unlocking its greater accuracy. Moreover, since QRNN models are effective at learning partial atomic charges, these models can accurately capture dynamics for polar and ionic systems, and condensed phase systems such as electrolytes^29^. As a result, the QRNN model holds promise to model polymer dynamics more accurately than classical force fields.

In this paper, we discuss the benefits of using the QRNN approach in simulating ethylene glycol (EG) and its oligomers inspired by earlier charge-based models^21, 25, 30^ and related works exploring the generalizability of machine-learned force fields to arbitrarily long polymer chains^31, 32^. EG-based solvents are used across various applications such as solvents^33^, paints^34^, antifreeze^35^, hydraulic brake fluids^36^, inks^37^, plastics^38^, films^39^, and cosmetics^40^. More recently, EG-based solvents have been used with ionic liquids^41^ and eutectic solvents^42^. Traditional biomolecular force fields, such as OPLS, have been used to model systems such as polymers^43^, ethylene oxide^44^, organic/inorganic nanostructures^45^ and ethylene glycol^46, 47^. This force field type has been successful in capturing some thermodynamic properties such as density at a constant temperature, however, it fails to capture some dynamic and thermodynamic properties such as self-diffusivity, viscosity, and specific heat^48^. The dynamic properties are predicted particularly poorly since the OPLS force fields result in an effective stiffening of the oligomers due to exaggerated torsional barriers, which becomes more pronounced as the chain length increases^49^. Quantum mechanics based charge-fitting can improve the performance of OPLS force field^50^ for specific systems, but this is not the focus of our study. Here, we use a QRNN model to mitigate the inaccuracies from the OPLS force field as well as the computational expense posed by traditional quantum computations. We describe the methodology for training force fields that may be applied to any class of polymers followed by a demonstration of their property prediction accuracy on ethylene glycol oligomers.

In this paper, we demonstrate the scalability of our model by training it on monomer, dimer, and trimer clusters and successfully applying it to oligomers ranging from tetramers to decamers. This scalability is a key feature of our model, justifying its characterization as “scalable”. Our simulations on longer polymer chains (> decamer) affirm the model’s ability to generalize to realistic polymer systems. Our hypothesis suggests that capturing the dynamics of non-conjugated systems can be achieved effectively by incorporating information from three monomers. However, it’s essential to note that the model developed in this study is not universally applicable to all polymer systems. To adapt it to a new polymer, it’s necessary to extract relevant clusters and include them in the training process. The advantage of our workflow lies in its support for transfer learning, obviating the need to train the model from scratch; instead, we can utilize the best-performing model from our current work as the initial input model.

Our research serves as a proof of concept, demonstrating the feasibility of employing our QRNN workflow for modeling polymer materials. It represents a successful and cost-effective approach for exploring the mechanical and thermophysical properties of polymers, ultimately enhancing our ability to design novel polymer systems with greater confidence.

The paper is structured as follows. In Section "Methods" we discuss the workflow for the machine-learned force field training set construction followed by the MD analysis specifics. Promising improvements to the neural network potential, such as carrying out feature engineering and active learning, are also discussed in this section. Following, in Section "Results" we discuss the numerical results obtained from the QRNN-MD simulations. We first show the results from the initial trained model (Section "Comparison of properties") and follow up with results from an improved model that implements feature engineering and active learning (Section "Model revisions"). We also discuss the consequence of such model improvements on the numerical results. We conclude the paper with the findings from our study and the promise that this scalable and generalizable force field provides.

## Methods

### Classical force field molecular dynamics

Given that polymers comprise repeating units of monomers, the goal is to use small oligomers ($\le$ trimers) to train the QRNN model, which then functions as the force field for the MD simulations for larger oligomers, which serves as the test of the model’s extensivity. We begin with developing a set of benchmark MD simulations, against which we will eventually compare the QRNN models performance. For the MD simulations, we use the OPLS4 force field developed by Schrödinger Inc^51^. We generate different simulation cells with increasing chain length, from pure monoethylene glycol to pure decaethlyene glycol. For creating amorphous cells, we use the Disordered System Builder (DSB) tool in Schrödinger Materials Science Suite (MSS), version 2022-2^52, 53^. The DSB tool arranges the oligomers in the simulation cell by rotating the backbone dihedrals, using a self-avoiding random walk algorithm, to ensure varying chain configurations. For each oligomer type, we create 10 replicate cells with different spatial configurations. The generated simulation cells consist of $\sim 1500$ atoms with a density of 0.5 g/cm$^3$. The initialized configuration is then relaxed in a ten-step process, as described in Section S1.1.

To obtain MD-generated values by using the OPLS4 force field, we performed a constant-temperature, constant-pressure ensemble (NPT) simulation on the relaxed configuration for 2 ns at 300 K and 1 atm external pressure with a timestep of 2 fs. We used Martina-Tobias-Klein (MTK) barostat and Nose–Hoover thermostat for all the OPLS4 simulations. The thermostat relaxation time constant was fixed at 1 ps whereas the time constant for the barostat was fixed at 2 ps. For the computation of self-diffusivity, we performed an a constant-temperature, constant-volume ensemble (NVT) simulation for 10 ns at 300 K with a timestep of 1 fs (since any ensemble with a barostat has the potential of interfering with the system dynamics). We use the mean-squared displacement and the Einstein equation to obtain the self-diffusivity.

The error bars shown in all of our results indicate the standard deviation among the ten replicates used for each system.

### Dataset preparation for QRNN training

From the OPLS4 trajectories, we extracted N-molecule clusters of monomers, dimers, and trimers of ethylene glycol to serve as training data. In the interest of efficient DFT labeling of the clusters, we limit our dataset to 8-molecule clusters in the case of the monomers, up to 6-molecule clusters in the case of the dimers, and up to 4-molecule clusters in the case of the trimers. We then sample several conformational clusters from the different N-molecule clusters where we find a balance between the number of clusters for representative training data and the computational expense associated with carrying out DFT calculations on the clusters. These conformational clusters contain non-equilibrium bond distances, and bond and dihedral angles which sample configurations that are representative of what the system might encounter during the course of a stable MD trajectory. The sampled conformational clusters also include density variations in addition to the bond, angle, and dihedral deformations. In addition to the sampled conformational clusters of a different number of molecules of the monomers, trimers, and dimers, we also look at a few decomposed samples of clusters. Decomposed samples are created by arbitrarily breaking/deleting bonds in our sampled configurations. These samples force the individual molecules into unfavorable configurations. The decomposed clusters prevent the QRNN model from stabilizing unrealistic molecular configurations while carrying out the MD simulation. The number of different N-molecule conformational samples from the regularly identified clusters is 210,500, whereas the total number of decomposed clusters at 106,000. The breakdown of the clusters is shown in Table S1. Out of these, a total of 217,684 clusters were used to compute the DFT energies, atomic forces, and dipoles. All the DFT data generated in this work is shared on figshare platform^54^. All DFT calculations were performed at the $\omega$B97X-D3BJ/def2-TZVPD level^27, 28, 55^ with the electronic structure software package Psi4-1.3^56^. We selected $\omega$B97X-D3BJ/def2-TZVPD level as it is a recommended theory from a benchmarking study by Grimme et al.^57^. The parameters used for the energy calculation were: 1e-10 DFT basis tolerance, 1e-10 Schwarz screening threshold, and 1e-6 linear dependency cutoff.

### QRNN training

The network used for the polymer force field predictor is taken to be the previously reported QRNN architecture^26^. Our study extends QRNN MD simulations to systems of ethylene glycol and its oligomers from monoethylene glycol through decaethylene glycol. We explore the extent of the restriction of the training space that would yield a reliable machine-learned force field for oligomers of any arbitrary length. To this end, we train three separate models: (i) QRNN - M: A model trained exclusively on the conformational and decomposed clusters of ethylene glycol, (ii) QRNN - M,D: A model trained exclusively on the conformational and decomposed clusters of ethylene glycol and diethylene glycol, and (iii) QRNN - M,D,T: A model trained exclusively on the conformational and decomposed clusters of ethylene glycol, diethylene glycol, and triethylene glycol.

The QRNN model is briefly summarized here, and readers interested in further details are referred to previous reports^26, 29^. QRNN is a modification of the HDNNP architecture designed to equally support ionic and neutral systems and describe long-range interactions with physics-based functional forms. Atomic neural networks are used to predict atomic electronegativities, using Behler-Parinello type symmetry functions^22^, or atomic environment variables (AEV) as input^16^. These predicted atomic electronegativities are then used in a simplified charge equilibration (Qeq) formula to determine atomic charges. As such, our models resemble the fourth-generation HDNNP architecture^21^. This charge prediction process is recursive, albeit limited to two iterations, which gives the model its name^26^. Once the charges have been determined, they are used as features, along with AEV and a charge-weighted radial AEV, to an atomic network that predicts energies. To compute the total energy, we sum the short-ranged neural network atomic energies, together with a long-ranged Coulomb energy (again using the predicted charges) and Grimme’s dispersion correction^58^ combined with the damping function proposed by Chai and Head-Gordon^59^. As a result, this model effectively captures long-range Coulomb and dispersion interactions while also representing short-range interactions with high precision.

As in previous works, we use a multitask loss function^60^, which describes the overall training error without having to hand-tune hyperparameters associated with weighting the individual tasks that comprise the loss function,

1 $$\begin{aligned} {\mathcal {L}}_{mtl} = \frac{{\mathcal {L}}_{E}}{2 \sigma _E^2} + \frac{{\mathcal {L}}_{g}}{2 \sigma _g^2} + \frac{{\mathcal {L}}_{q}}{2 \sigma _q^2} + log(\sigma _E \sigma _g \sigma _q) \ . \end{aligned}$$

Here, ${\mathcal {L}}_E$ is the squared error in energies, ${\mathcal {L}}_g$ denotes the squared error in energy gradients, ${\mathcal {L}}_q$ is a loss function for charges, and $\sigma _E, \sigma _g, \sigma _q$ are trainable parameters. While a loss function for charges is not strictly required if the training set adequately covers long-range interactions, it can guide the model to provide accurate atomic charge predictions, which in turn yield accurate long-range energy predictions. In previous work, we utilized a charge loss function that quantified the error in predicted dipole moments^26^. The rationale behind this approach is that for a neutral molecule, the dipole moment is the leading order in a multipole expansion, and reproducing the dipole ought to yield reasonable reproduction of the long-range electrostatic potential. Moreover, the dipole moment is also an exact property of the electron density. For larger molecules, however, the dipole moment provides limited information about the distribution of atomic charges, similar to how the total energy provides less information than forces. Further, molecular symmetry may enforce a zero dipole moment, yielding no information that most MLFF models won’t trivially reproduce. Therefore, it is useful to supply reference atomic charges and consider ${\mathcal {L}}_q$ as the squared error of these atomic charges. In this work, we investigate the use of atomic charges from GFN2-xTB as they can be efficiently computed for existing data, and the GFN suite of methods yields good reproduction of intermolecular energies and molecular dipole moments^61^.

For training, we use weight normalization with a weight decay of $10^{-4}$. The learning rate ($\alpha$) is set at $10^{-3}$. The training batch size is 256, while the validation batch size is 1024. We use 500 epochs with an epoch size of 200,000 samples during training. The training labels we use initially are total energy, atomic forces, and dipole moments. For some models (described later in the paper), we replace the dipole moments as with extended tight-binding charges as a training label.

### QRNN aided molecular dynamics

The molecular dynamics simulation from the QRNN force field was performed using the Atomic Simulation Environment (ASE) package with the QRNN being used as a force calculator. For all the QRNN MD simulations, the total time of the simulation is 1 ns with a timestep of 0.5 fs owing to the computational expense and numerical stability associated with the simulation. The Nose–Hoover thermostat was used for the NVT and NPT simulations and the Parinello-Rahman barostat was used for the NPT simulations. The thermostat relaxation time constant was fixed at 50 fs whereas the time constant for the barostat was fixed at 2.5 ps. The QRNN NPT simulations are run starting from the equilibrated structure obtained from the relaxation protocol mentioned in Section S1.1 which utilizes the OPLS4 force field. We used all ten simulation boxes (replicates) for each of the ten oligomers of ethylene glycol that were equilibrated using the OPLS4 force field. For the case of the NVT simulation, we use a snapshot from the QRNN-MD trajectory that has a density that is close to the average density of the NPT trajectory. The NPT and NVT simulations are run for a total of 1 ns. For the computation of self-diffusivity, we sample the trajectory every 0.5 ps.

To test the transferability of the QRNN model to longer polymer chains, we conducted simulations for chains consisting of 25, 50, and 100 monomers in addition to the ten oligomers. We generated ten simulation boxes for each chain length, containing approximately 8000 atoms. The QRNN simulations were run using the Desmond implementation on a GPU with 3584 CUDA Cores for 5ns, with a time step of 1fs

### Model validation

In a conventional validation process of a machine-learned force field, predicted values are typically compared with DFT predicted values for a few selected clusters. We have performed such validation in our work and report parity plots for energies, forces, and charges, to show the agreement between predicted and DFT values. In addition to these parity plots, we introduce the pair scanning method as an extra means of validating our models. This pair-scanning approach is incredibly powerful as it allows us to visualize the quality of our models in a more comprehensive manner. To conduct the pair scanning, we randomly select a pair of neighboring molecules from the QRNN MD simulation and run the energy calculation on just these two molecules with increasing separation distance. As these pairs are extracted from MD, they are close to the most optimal distance from each other. To obtain the potential energy curve showing this optimal distance, one of the molecules is manually translated along the line of the center of mass of the two molecules. This process constitutes of rigidly translating one of the molecules from a distance of $d_{{min}}$ to $d_{{max}}$ in $n_{\text{steps}}$, where $d_{{min}}$ is chosen to be about $75\%$ of the initial distance between the center-of-mass of the two molecules ($d_{\text{COM}}$) and $d_{{max}}$ is chosen to be $300\%$ of $d_\text{COM}$. For these $n_{\text{steps}}$ configurations, we compute the potential energy of the 2-molecule system using DFT, the OPLS4 force field, and the QRNN force field. For well-trained models, we would expect the energy-separation landscape from the QRNN model to be much closer to the DFT results. For our analysis, we use $n_\text{steps} = 500$ for ethylene glycol, diethylene glycol, and triethylene glycol. However, for tetraethylene glycol we use $n_\text{steps}=300$ in the interest of computational expense.

### Active learning

To further improve the QRNN MD accuracy, we added additional DFT samples. We do this efficiently by implementing an active learning method, i.e., to smartly select the samples that would improve the model accuracy and remove redundant samples. Active learning constitutes a multi-step process:

- We first train an ensemble (also known as a committee) of five QRNN models by using the original dataset that we wish to improve. However, instead of using a 90-10 split for training-validation sets (as we do for training the target model), we use a 60-40 split to introduce more variation between the models.
- We follow this step by creating a large number of clusters of a different number of molecules for the monomer, dimer, and trimers of ethylene glycol. In our particular case, we create close to 800,000 clusters for the first round of active learning, details of which are shown in Table S2.
- We use all five models to compute the atomic forces across clusters and note the weight-averaged variance ($\rho ^f$) of the computed forces for all the $N_a$ atoms in each cluster given by,
  2 $$\begin{aligned} \rho ^f = \sum _{i=1}^{N_a}\sum _{x,\,y,\,z} \sigma (f)/\sqrt{N_a}. \end{aligned}$$
  Here, $\sigma (f)$ is the variance of forces computed by five models for a single atom and $\rho ^f$ serves as a measure of difficulty in generalizing the force field to a given cluster sample.
- We select 40,000 samples with the highest $\rho ^f$ and another 40,000 samples that are randomly selected from the remaining conformational clusters. To this, we add the newly extracted clusters (Cluster column in Table S2) to form the active learning samples.
- The DFT calculations are now run on the active learning samples after which they are appended to the database.
- A new QRNN model is trained with these additional samples in the database.

We follow the same steps for the second round of active learning with a few differences. The total number of clusters extracted from our QRNN MD trajectory for the second round is $\sim 313,000$ and does not include any conformational samples. We perform the $\rho ^f$ calculations directly on the extracted clusters. From this, we select 30,000 samples with the highest $\rho ^f$ and another 30,000 samples that are randomly selected from the remaining extracted clusters. The details of the extracted clusters used for the second round of active learning can be found in Table S3.

## Results

We compare the results obtained from the OPLS4 force field and the machine-learned force field (QRNN) against the experimental results for ethylene glycol oligomers. The OPLS4 MD simulation results carry with them higher inaccuracies in capturing certain dynamic and thermodynamic properties due to the insufficient accuracy of the force field. Part of the inaccuracy can be attributed to the size of the systems we are working with ($\sim 1500$ atoms), which is the scale limitations imposed by the QRNN-MD model that we are comparing the OPLS4 results against. However, we demonstrate how the QRNN force field captures atomic interactions more accurately, requiring a much smaller atomic system to give comparable/better/more converged results than the OPLS4-MD obtained simulations. The experimental values for all physical and chemical properties across all oligomers are not available at 300 K, as a result of which we interpolate the values of density and self-diffusivity obtained for ethylene glycol and its oligomers at 298.15 K and 303.15 K by^62^. All models described in this section perform well on the validation dataset with the predicted energy being well correlated with the reference energy (see Fig. S1 for parity plot from the best performing model). No explicit comparisons of the QRNN model’s performance is made against graph-based methods since they are computationally expensive and memory intensive with a simulation throughput which is significantly slower than the QRNN model’s throughput.

### Pair scanning

**Figure 1:**
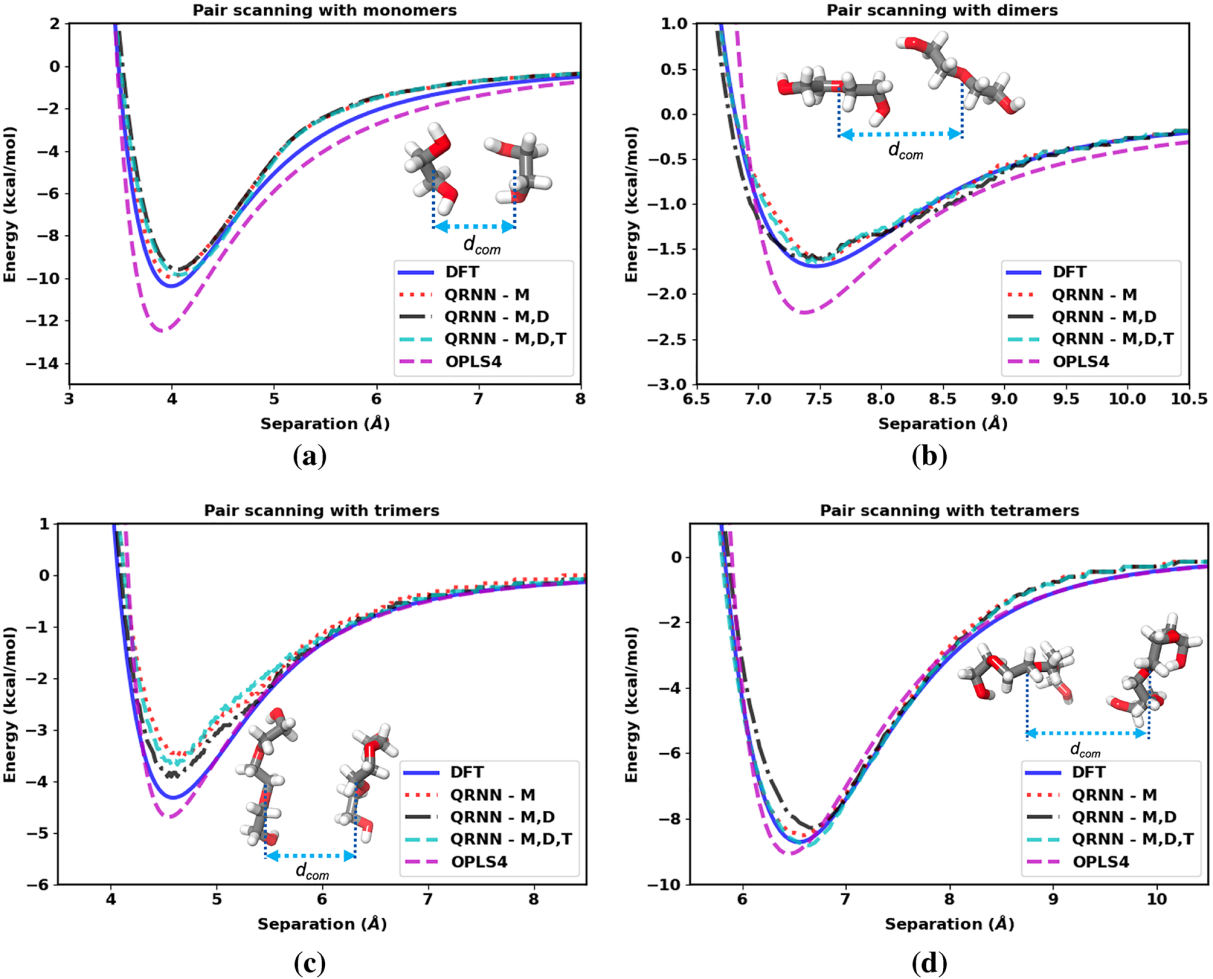
Separation energy between two molecules as a function of separation distance compared between the DFT calculation, OPLS4 force field, and the machine-learned force field for the (**a**) monomers, (**b**) dimers, (**c**) trimers, and (**d**) tetramers of ethylene glycol.

As stated earlier, pair scanning provides a measure of the performance of a force field. In this case, the ground truth for the interaction energy is considered to be the energy computed from the DFT calculations carried out using $\omega$B97X-D3BJ^27, 28^ functional and the def2-TZVPD^55^ basis set with the electronic structure software package Psi4-1.3^56^, just as the protocol followed for dataset preparation. The hypothesis is that the machine-learned force field should do much better than the OPLS4 force field since it is trained on the DFT data that we are benchmarking the pair scanning results against. To illustrate our argument for the model accuracy, we test all three models: QRNN - M, QRNN - M,D and QRNN - M,D,T for monomers, dimers, trimers, and tetramers, as shown in Fig. 1.

We see that the force field that is trained on just the monomers generalizes equally well to oligomers up to the size of tetraethylene glycol. We see that the machine-learned force fields do better than the OPLS4 force field in estimating the energy near the optimal pair separation, which indicates that the QRNN models are likely to capture the dynamics better than the OPLS4 force field. We computed the gradient of the tetramer pair (see Fig. S2), which shows that the force to separate the molecules is higher for OPLS4 when compared to DFT and QRNN models. Even though the largest molecules in the clusters used for training were restricted to triethylene glycol, all the models do reasonably well on predicting the interactions of tetraethylene glycol. This test corroborates the transferability and generalizability of the model to oligomers of lengths longer than the oligomers comprising the training dataset. However, a telling feature of the pair scanning is that the energy minima with the QRNN models appear to the right of the DFT calculations, which implies that the QRNN model places the clusters at a larger equilibrium distance apart. As a result, the density prediction from the QRNN models is expected to be lower. The peak position is a proxy for the density prediction, whereas the gradients of the energy landscape (the resultant force) can be used as a proxy for the feature that captures the dynamics. Our goal would be to sample configurations such that we can shift the QRNN energy landscape to match the DFT energy landscape to get the correct density and dynamics.

### Comparison of properties

#### Density

**Figure 2:**
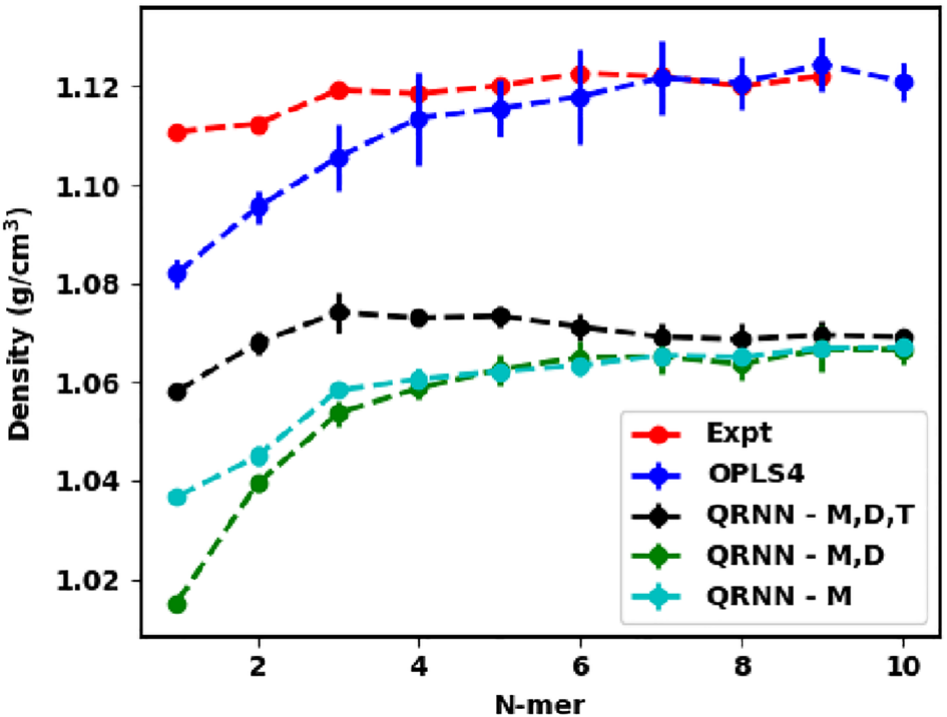
Predicted density from the OPLS4 force field and the machine-learned force field compared against the experimental density.

The Lennard-Jones intermolecular interactions of the OPLS family of force fields are fitted to reproduce experimental properties, so it is not a surprise that the OPLS4 force field is known to capture the density of ethylene glycol oligomer systems within 1$\%$^63, 64^. We see that the OPLS4 force field predicted density is still within $1\%$ and is strongly correlated with the experimentally obtained values for the oligomers in our analysis, as shown in Fig. 2. On the other hand, we see that the machine-learned force fields underpredict the density by $~5\%$. Note, that the density from the QRNN MD is averaged over the last 800 ps of the 1 ns simulation, to avoid convergence problems.

Experimentally, we see the density values converging to 1.12 g/cm$^3$^62^ as the oligomer length of ethylene glycol increases. This trend is accurately captured by the OPLS4 force field where the density prediction becomes closer to the experimental values with the increase in the oligomer length. The density prediction from the QRNN models is still successful in capturing the convergent density prediction, although it happens to be lower than the expected density. This highlights that the QRNN model can be trained on smaller constituent molecules of a polymer to run simulations on larger polymer chains due to the generalizability of the HDNNP.

#### Self-diffusivity

**Figure 3:**
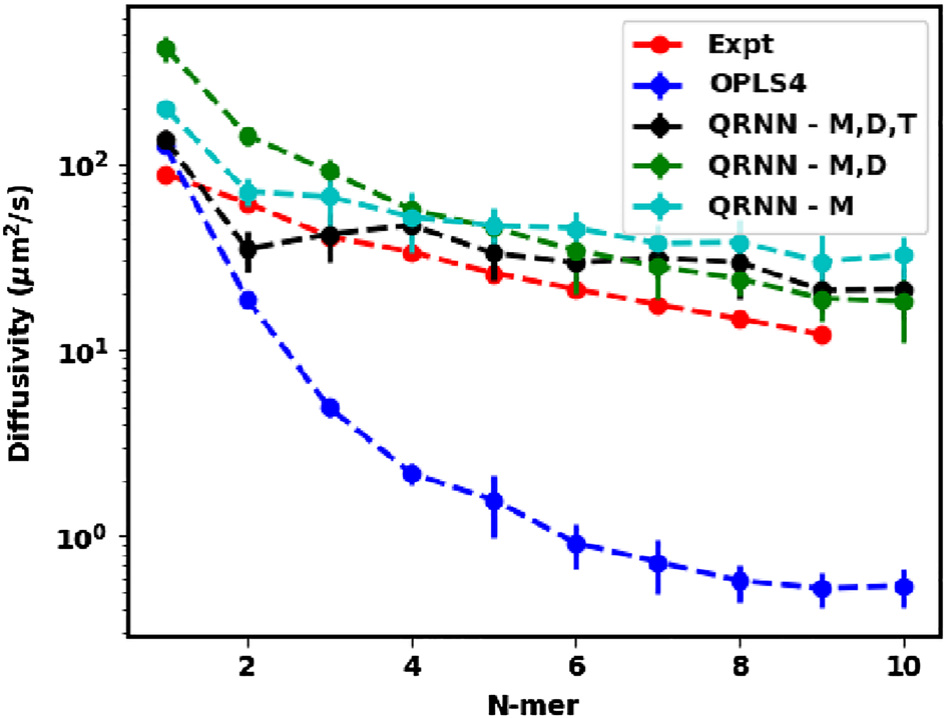
Predicted diffusivity from the OPLS4 force field and the machine-learned force field compared against the experimental diffusivity.

The OPLS4 force field has its drawbacks in simulating the dynamics of polymeric networks. Typically, longer oligomers are artificially stiff using the OPLS4 force field, as shown in Fig. 3. For the monomer ethylene glycol, we see that the measured diffusivity is in the same order of magnitude as the experimentally observed self-diffusivity. However, for oligomers starting as small as the dimer, we see a sharp drop-off in the self-diffusivity. This rigid locking of the oligomers might result in them getting trapped in solid-like configurations.

On the other hand, we see that the QRNN force field does not reduce the larger oligomers to such configurational locking allowing for a more diffusive trajectory during the simulation. However, the self-diffusivity from the QRNN models overpredicts the experimental self-diffusivity. This observation can be attributed to the lower density configurations that are generated by the QRNN MD simulation. The lower density affords the oligomers an ease in migration within the simulation box resulting in the prediction of a higher self-diffusivity.

#### Specific heat capacity

**Figure 4:**
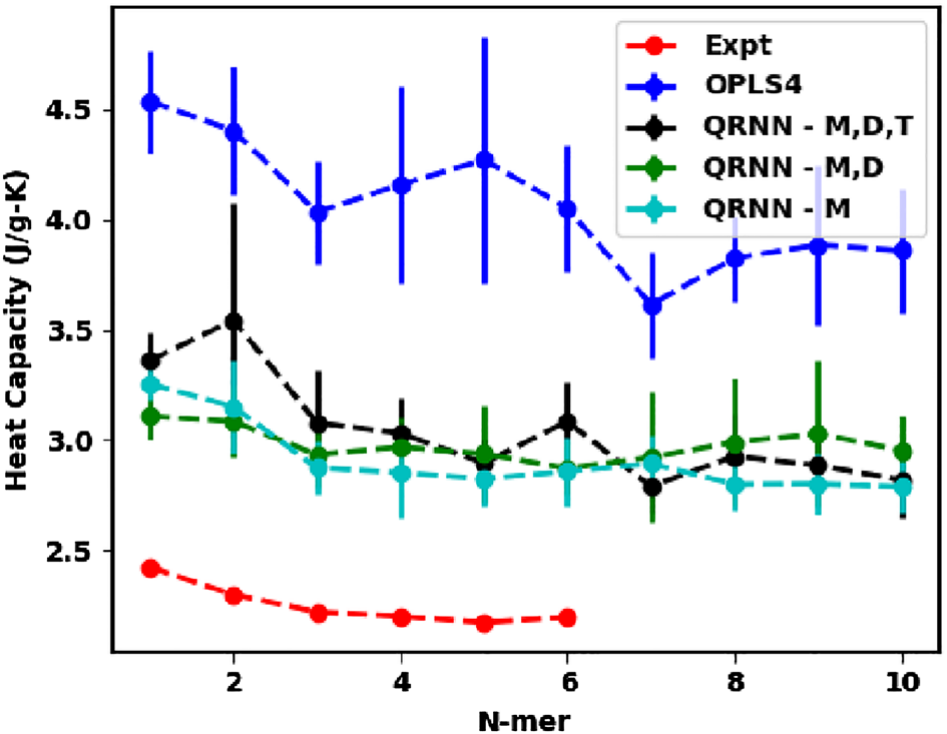
Predicted specific heat capacity from the OPLS4 force field and the machine-learned force field compared against the experimental specific heat capacity.

The specific heat capacity for molecular simulations is computed by using the fluctuation-dissipation theorem, where the specific heat at constant pressure, $c_p$, is given by,

3 $$\begin{aligned} c_p = \frac{\langle E^2\rangle _{\text{NPT}}-\langle E\rangle ^2_\text{NPT}}{k_BT^2}, \end{aligned}$$

where *E* is the total energy, $k_B$ is the Boltzmann constant and *T* is the temperature held by a thermostat in the NPT simulation. For ethylene glycol and its oligomers, we have observed a consistent overprediction of the $c_p$, which is commonly observed for the OPLS force field without quantum corrections^48^, as confirmed by our simulations (see Fig. 4). For our QRNN model trained on *ab initio* calculations, we expect the energy fluctuations to be captured better than the traditional OPLS4 force field. For all three models that we have implemented, we see that the $c_p$ predictions are much closer to the experimentally observed values compared to OPLS4, even though they are still overestimated. The overprediction in the $c_p$ values is due to nuclear quantum effects (NQE). This overprediction could be overcome by implementing quantum corrections as originally described by Berens et al.^65^, but we do not account for such corrections in the current work.

### Model revisions

The machine-learned force field showed promising results. The MD trajectory was stable across all replicates, however, the density predictions are not as close to the experimental values as the OPLS4 force field predictions are. The heat capacity and diffusivity predictions are better than the OPLS4 force field, however, they are still not in agreement with the experimentally obtained values. The recurring inference to all our results was that the mismatch in the density predictions could all be stemming from the low-density configurations that the QRNN MD simulation was running into. To address this density mismatch we follow a two-pronged approach to improve our force field: (i) add samples to the dataset by doing active learning, and (ii) train the model on extended tight binding (GFN2-xTB, hereby referred to as xTB) charges instead of the dipole moment.

For active learning, we follow the multi-step protocol described in the Section "Active learning". The $\rho ^f$ is expected to be directly correlated to the energy prediction error, $\epsilon = | E_{\text{DFT}} - E_{\text{QRNN}} |$.

**Figure 5:**
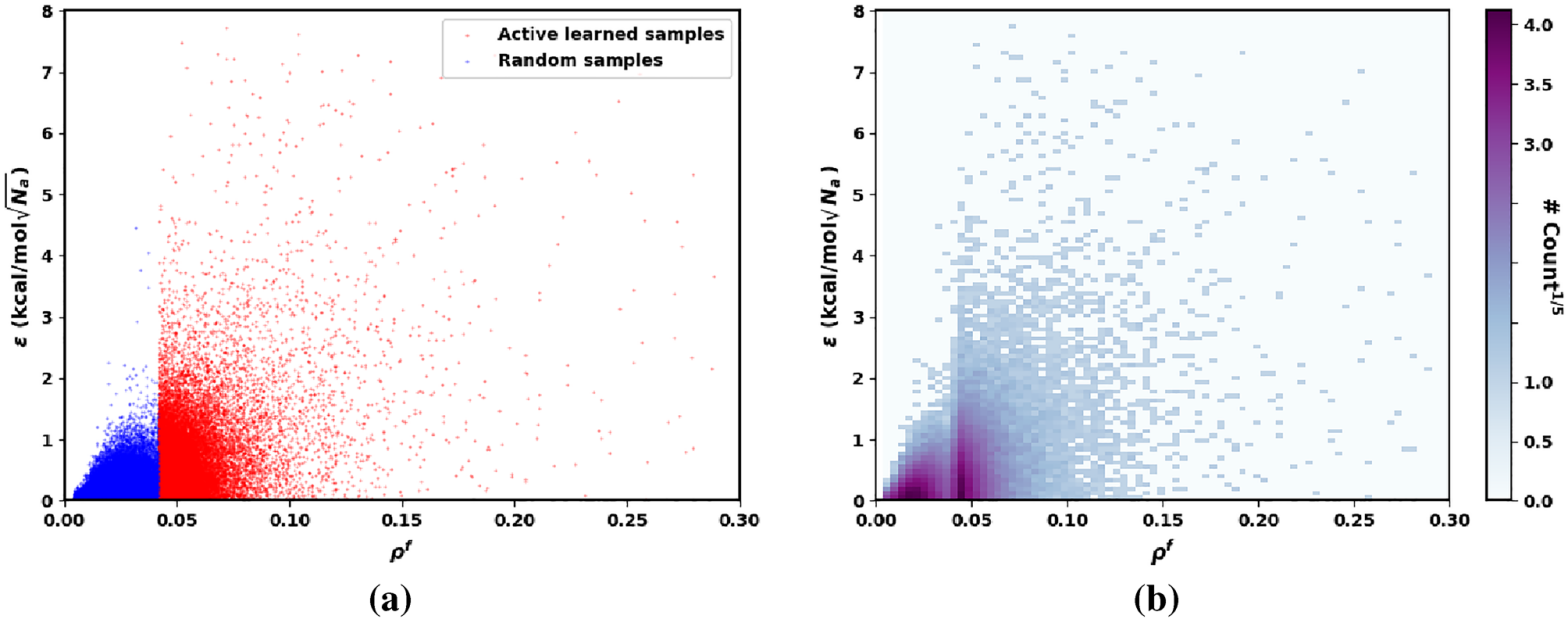
(**a**) Location of the active learned samples (red) and random samples (blue) on the $\epsilon$ vs $\rho ^f$ landscape. (**b**) Heat map (by $\#$counts$^{1/5}$) of the $\epsilon$ vs $\rho ^f$ landscape for the conformational samples selected for retraining.

We plot the distribution of the active learned samples (red points) and random samples (blue points) in Fig. 5a, while Fig. 5b shows the count of the selected samples in a specific region on the $\epsilon$ vs $\rho ^f$ landscape. We observe that randomly selected samples tend to have a higher frequency of low $\rho ^f$, which explains why the model has not generalized well to high variance clusters of active learned samples. By incorporating these samples into the training, significant improvement in the model is expected, bridging the density prediction mismatch.

The default workflow is designed to train the network (one network per atom type) to learn the total energy, the forces per degree of freedom (from the backpropagation gradient of the energy landscape), and the dipole moment. As an alternative feature engineering approach, we can replace the dipole moment as a feature with the xTB charges. This can be a helpful modification since ethylene glycol and its oligomers are symmetric as a result of which they have insignificant dipole moments. This makes the weights corresponding to the dipole moment label difficult to train. To this end, the network could be trained on the atomic partial charges, but the DFT-derived calculations, based on population analysis, are sensitive to the choice of the density functional basis set. However, xTB charges are more accurate and depend only on the local interactions imposed by the tight-binding approximation^61^. Hence, xTB charges as a label provide a solution since the computed partial charges will not be diminishingly small.

For brevity and to demonstrate the incremental improvement shown by active learning and the replacement of dipole moment as a training label with xTB charges, we present two models from the two rounds of active learning and compare them against QRNN - M,D,T. The first model (QRNN - AL-TL) is developed by carrying out transfer learning on QRNN - M,D,T by using the composite database at the end of the first active learning cycle (comprising the original database appended with the samples from the first round of active learning). The second model (QRNN - AL2.0-TL-xTB) uses the composite database at the end of the second active learning cycle and it transfer learns from a model that is trained on xTB charge labels (QRNN - AL-TL-xTB, a first active learning cycle model that is briefly described in Section S1.3). With both the newly trained models, we carry out the same analysis as before: (i) We use the NPT trajectory to compute the density and the specific heat capacity; (ii) we use the NPT snapshot closest to the average density over the NPT run as the starting configuration for the NVT simulation – the resulting trajectory is used to compute diffusivity and finally (iii) we carry out pair scanning on the monomer through tetramer of ethylene glycol to check the model performance.

**Figure 6:**
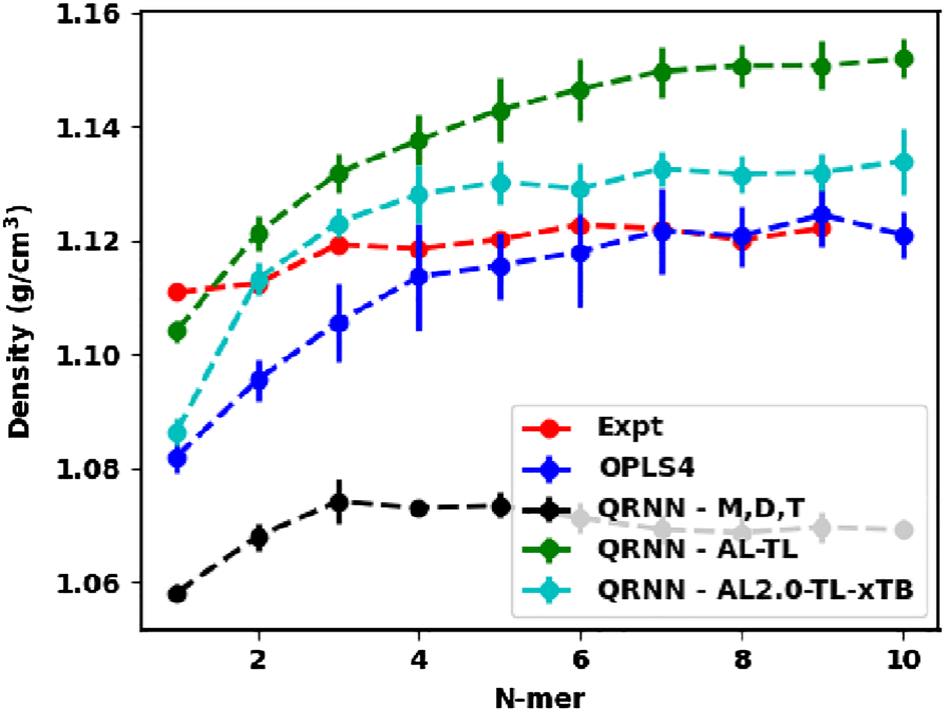
Predicted density from the OPLS4 force field and the machine-learned force field compared against the experimental density.

The model trained on the initial clusters resulted in under-predicting the experimental density. On carrying out transfer learning on the initial model with active learning samples appended to the original dataset, we see that the model, QRNN - AL-TL, now shows an overprediction of the density as seen in Fig. 6. We see that the active learned model underpredicts the density for ethylene glycol before transitioning to over-predicting the density for longer oligomers. The density from this new model eventually converges to  1.15 g/cm$^3$, which is an overprediction by $~3\%$. However, we see that QRNN - AL2.0-TL-xTB, model from the second round of active learning, circumvents this shortcoming with an overprediction of the density by under $1\%$. This shows that multiple rounds of active learning could help the model eventually converge to the experimental density, which appears to be the mean of the previous model and the newer model with the active learned samples. We also note that the model trained on the xTB charges instead of the dipole moments provides a more convergent density and shows a very good correlation ($R^2=0.81$ – see Fig. S10) with the experimentally observed density. We observe that the fitted force field underpredicts the density of the monomer. This is likely because most of the selected training samples in the active learning phase belonged to dimer and trimer samples. Consequently, the final model may not have been adequately trained for monomer samples. Our primary objective was obtaining an improved model for the polymer limit; thus, we did not prioritize optimization for monomers.

**Figure 7:**
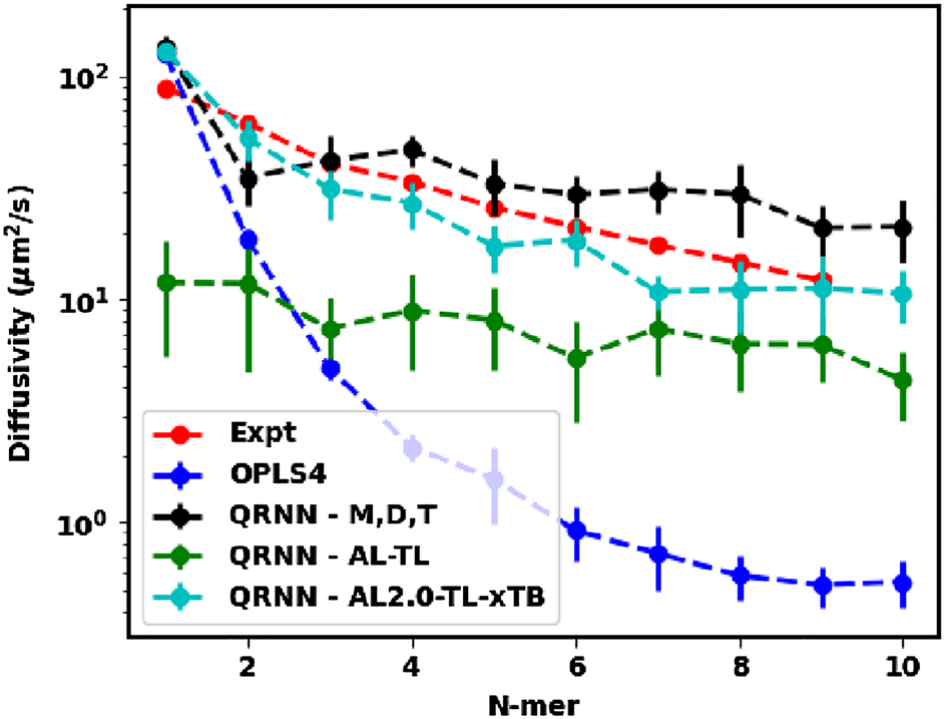
Predicted diffusivity from the OPLS4 force field and the machine-learned force field compared against the experimental diffusivity.

Dynamics prediction appears to be the bottleneck associated with the OPLS4 force field as well as our initial set of models. Even though our model circumvents the artificial locking/freezing of longer oligomers that the OPLS4 force field induces we still observe overprediction by 40–80%. With higher densities being predicted by the two new test models, we expect to see a lower self-diffusivity, which is corroborated by our results shown in Fig. 7. We see that QRNN - AL-TL shows an underprediction of the self-diffusivity from the experimental values by about $40-60$. The model trained on the xTB charges, QRNN - AL2.0-TL-xTB, at the end of the second active learning cycle performs much better in the prediction of the diffusivity and it most accurately captures the drop in diffusivity with the oligomer length (within $30\%$), showing the best correlation with the experimental self-diffusivity ($R^2=0.91$ – see Fig. S11).

**Figure 8:**
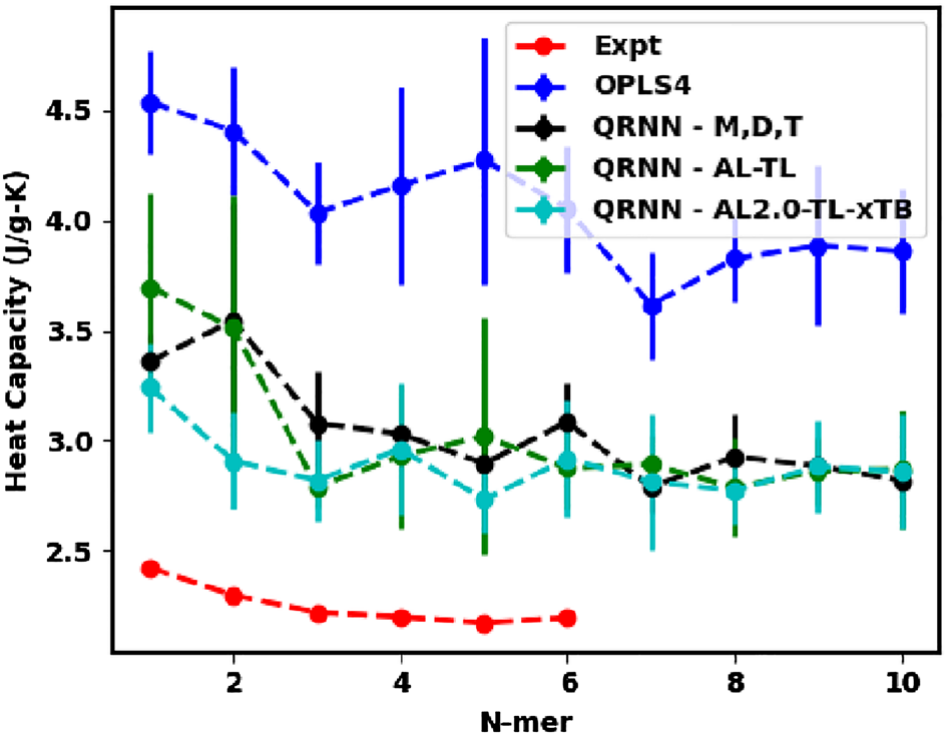
Predicted specific heat capacity from the OPLS4 force field and the machine-learned force field compared against the experimental specific heat capacity.

The increase in density that is enforced by the modified force field results in the slowing of the dynamics. We can expect the variance in the total energy to be limited since a major contribution to the energy fluctuations is brought about by the kinetic energy contribution. Our hypothesis is corroborated by the specific heat capacity calculations in Fig. 8. Additionally, we see that the QRNN - AL-TL and QRNN - AL2.0-TL-xTB models predict the $c_p$, which is much more correlated to the experimental values than the OPLS4 force field or the initial QRNN - M,D,T model. Furthermore, the $c_p$ prediction made by the QRNN - AL2.0-TL-xTB is better for smaller oligomers before converging to the long chain $c_p$ predicted by the other trained models.

**Figure 9:**
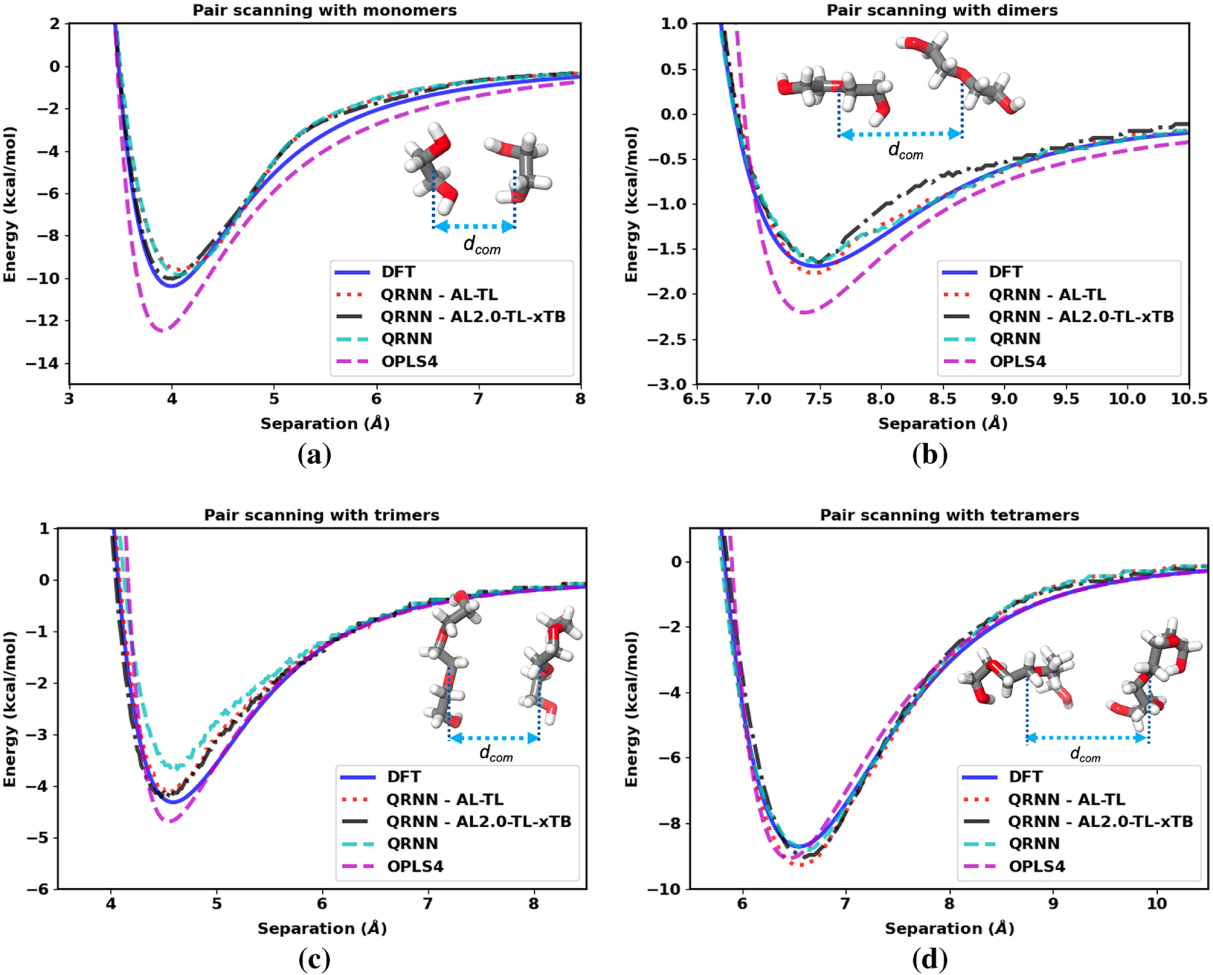
Separation energy between two molecules as a function of separation distance compared between the DFT calculation, OPLS4 force field, and the machine-learned force field for the (**a**) monomers, (**b**) dimers, (**c**) trimers and (**d**) tetramers of ethylene glycol.

As a final test for the quality of the force field that is newly trained, we carry out the pair scanning test. The QRNN models are qualitatively similar in their performance across oligomers of all sizes, as shown in Fig. 9. Much like the previous analysis, the QRNN models perform better than the OPLS4 force field in terms of force prediction. In addition, we also see a left shift in the energy well obtained from the QRNN models after the proposed changes to the training protocol (energy well from the red dotted lines and the black dash-dot lines is to the left of that of the blue dashed line which represents the initial model), implying higher density. The eventual goal for any material system would be to train a model through iterative active learning such that the energy well from the DFT calculations and the QRNN model overlap for the most accurate density and dynamics prediction.

The behavior of polymer systems is greatly influenced by the accuracy of our consideration of torsion energies. To assess this accuracy, we conducted an energy analysis by systematically rotating the dihedral angle of the backbone. This investigation was carried out using the ethylene glycol dimer, with a focus on the rotation of the OCCO bond. Our energy calculations were performed utilizing three different methods: DFT, QRNN, and OPLS4.

**Figure 10:**
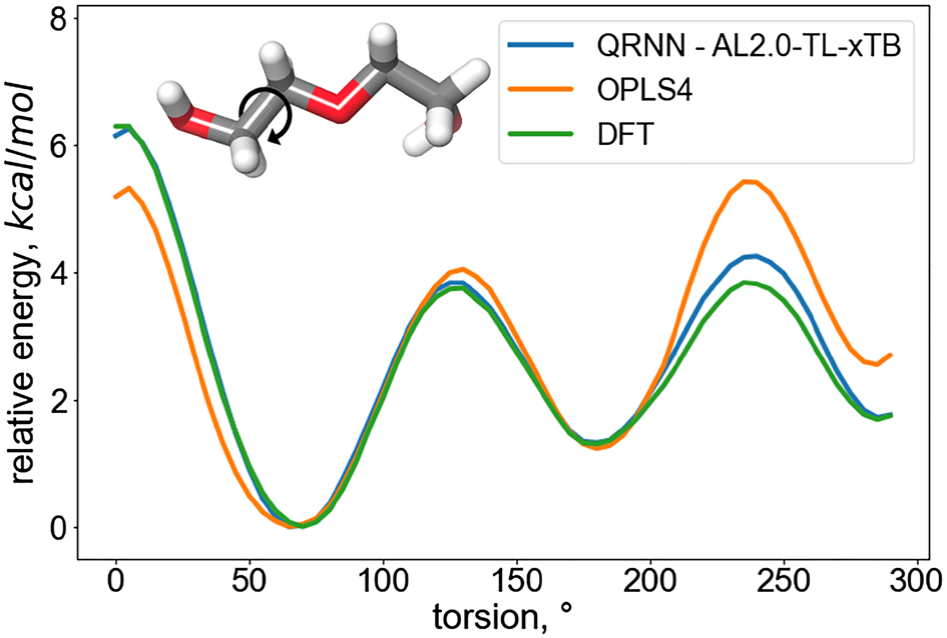
Relative energies and energy barriers for the torsion scans performed (5$^{\circ }$ steps) on OCCO dihedral.

Figure 10 illustrates a comparison of the torsion energy results obtained from all three methods. Notably, our findings reveal that the QRNN method closely aligns with the DFT results, while OPLS4 tends to overestimate the energy barriers associated with the initial two rotations. These outcomes suggest that OPLS4 yields a stiffer polymer chain compared to QRNN, which, in turn, leads to lower diffusion values for OPLS4.

### Model summary and extensibility

From these results, we conclude that both our strategies, (i) active learning and (ii) active learning with xTB labels, were successful in achieving our desired behavior of increasing the density predictions. The first learning cycle resulted in the density under-prediction transitioning to density over-prediction. This trend is then improved by the second active learning cycle, which demonstrates the success of active learning in nudging the model toward ideal behavior. As a result, we can draw insights on how to smartly sample configurations initially to overcome the issue with the under or overprediction of densities and the subsequent dynamics that arise from it. Carrying out multiple active learning rounds or smartly managing the distribution of the samples during active learning can help arrive at a convergent force field in fewer active learning iterations, as demonstrated by the incremental model improvement shown in Fig. S13. Since the diffusivity and the specific heat capacity are tied to the dynamics governed by the simulation box of a specific density, we can hypothesize that these properties will be predicted better once we arrive at a more convergent machine-learned force field.

**Figure 11:**
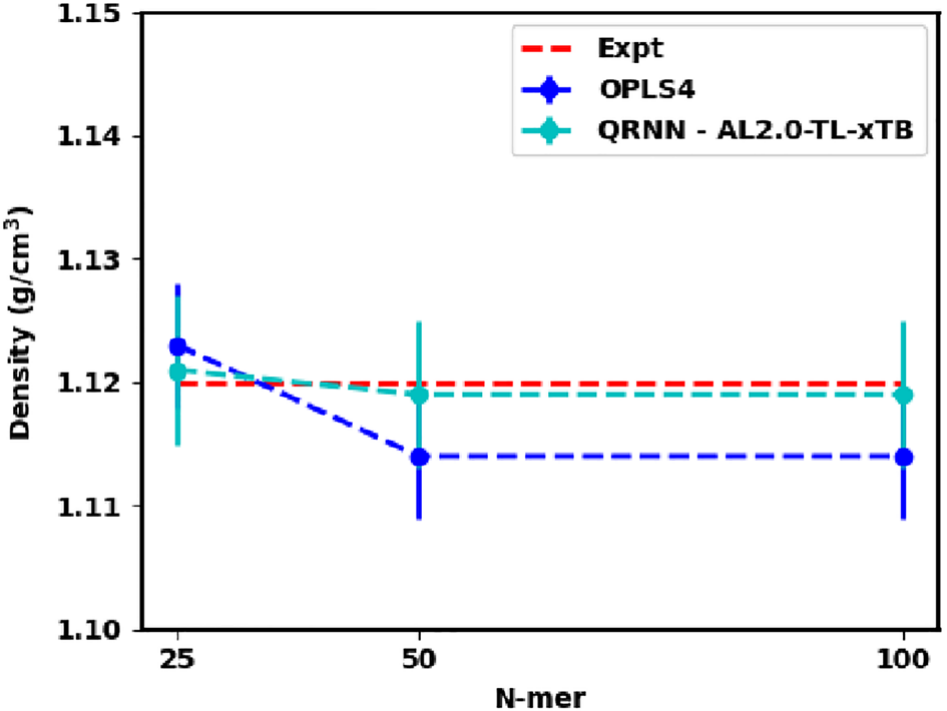
Predicted density from the OPLS4 force field and the machine-learned force field compared against the experimental density^66^

To further assess the stability and performance of our developed QRNN model, we applied it to polyethylene glycol chains of varying lengths, specifically with 25, 50, and 100 monomers. The outcomes of these simulations demonstrated consistent stability, as illustrated in Fig. S14a. We compared the computed densities of these systems to the extrapolated experimental density data^66^, as presented in Fig. 11. Our observations indicate that the QRNN method aligns well with experimental values, suggesting that the model, initially trained on monomer, dimer, and trimer samples, performs effectively on longer polymer chains.

**Figure 12:**
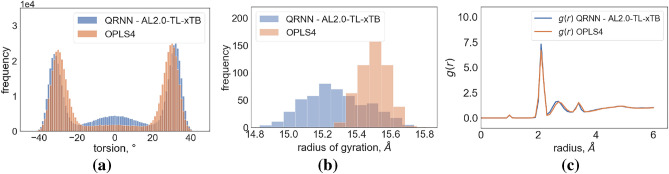
(**a**) Comparative Analysis of Torsion Angle Distributions: This figure presents a comparison of the torsion angle distribution from the OCCO dihedral of the backbone in the 100-mer chain, utilizing the last 500 frames obtained from both QRNN and OPLS4 simulations; (**b**) Radius of Gyration (Rg) Distribution of Chains: This figure shows the Rg of first chain from 100-mer system simulation using OPLS4 and QRNN methods; (**c**) Radial Distribution Function (RDF) Assessment of O–H Interactions: This figure illustrates RDF plots characterizing the interactions between O–H pairs in simulations conducted with both the QRNN and OPLS4 methods.

Additionally, we investigated the torsion angle distribution in the backbone of 100-mer systems, employing both the OPLS4 and QRNN methods (see Fig. 12a). This analysis aimed to assess whether the trends observed in Fig. 10 extended to the 100-mer chain. Notably, we observed a greater diversity of configurations between the two peaks in the QRNN method, indicating a higher probability of transitioning between these states. This results in a chain that exhibits greater flexibility compared to OPLS4 simulations. To further corroborate this observation, we examined the distribution of the radius of gyration (Rg) for a single chain within the 100-mer system simulation. As depicted in Fig. 12b, the Rg variation for the 100-mer chain in the OPLS4 method was found to be smaller, indicating a higher degree of rigidity in the OPLS4 simulation. Our analysis across various chain lengths, including 25-mers, 50-mers, and 100-mers, revealed that the QRNN methodology consistently yielded four times the variance in Rg compared to the OPLS4 methodology. The combined results from torsion angle distribution and Rg analysis support the conclusion that the OPLS4 gives stiffer molecular chains in comparison to QRNN.

In an effort to discern any variations in O–H interactions, we computed radial distribution function (RDF) plots (see Fig. 12c). Interestingly, our analysis revealed similar O–H radial distributions for both the QRNN and OPLS4 methods. Consequently, we can attribute the primary source of rigidity in the OPLS4 chain to the stiff torsional behavior of its backbone.

## Conclusion

In this paper, we present the development and performance of a QRNN force field for ethylene glycol and its oligomers by comparing it against results from experiments and the OPLS4 force field. The QRNN model is trained and validated against a reference level DFT theory to address the shortcomings of the OPLS4 force field in capturing the dynamic and thermodynamic properties of polymeric systems, such as self-diffusivity and heat capacity. The QRNN force field performs marginally poorer than the OPLS4 force field in estimating the density; however, it performs much better in capturing the specific heat capacity and the self-diffusivity. We see promising results from the QRNN force field in which the model trained on small oligomers generalizes well to longer oligomers.

Our QRNN model is trained only to small ethylene glycol oligomers, but generalizes well to longer oligomers - the QRNN model does relatively well in capturing the density, self-diffusivity, and specific heat capacity of the oligomer up to decaethylene glycol. Due to the superior quasi-linear scaling of the QRNN model^67^, compared to the cubic scaling of *ab initio* calculations, we are able to run simulations of systems of $\sim 8000$ atoms, where the QRNN model calculations (throughput of $\sim 2.7$ ns/day) are six orders faster than AIMD simulations (throughput of 1 fs/day). In comparison, the OPLS MD simulations (throughput of $\sim 550$ ns/day) are two orders faster than the present capability of our QRNN model. However, we demonstrate that the QRNN force field agrees better with ab-initio calculations which will allow us to capture processes and dynamics that a larger or longer OPLS MD simulation might not even be able to capture due to the shortcomings of the traditional force field.

To further improve the force field in the area of dynamics and the system density, we carry out two approaches: active learning and training on xTB charges instead of the dipole moment. We see that these approaches have the desired effect of incrementally improving the density and dynamics prediction of the machine-learned force fields. A smarter implementation of active learning by carefully including sampled configurations helps in improving the QRNN model, as demonstrated by this paper. Our work shows that training generalizable models for polymer systems is possible even with smaller building blocks such as monomers, dimers, and trimers. We also show that with a smarter training protocol and conformational sampling to cover the configuration space, we can develop a force field that is more adept than the classical force field at capturing the dynamics of the target system.

This approach paves the way for developing QRNN force fields for polymeric systems with limited computational expense (by training on smaller oligomers), which can generalize well to polymers of arbitrary length. As a result, we can now assess rare events and dynamical phenomena in such systems that were earlier inaccessible by traditional force fields (due to force-field inaccuracy) and ab-initio methods (due to length-scale limitations) – two problems that are well addressed by the QRNN force field.

### Supplementary Information

Supplementary Information.

## Data Availability

All the DFT data generated in this work is available on the figshare platform^54^. We provide additional data on cluster sampling and modeling methods in the Supplementary appendices.

## References

[1] Schmidt Jonathan, Marques Mário RG, Botti Silvana, Marques Miguel AL (2019). Recent advances and applications of machine learning in solid-state materials science. NPJ Comput. Mater..

[2] Wei Jing, Chu Xuan, Sun Xiang-Yu, Kun Xu, Deng Hui-Xiong, Chen Jigen, Wei Zhongming, Lei Ming (2019). Machine learning in materials science. InfoMat.

[3] George Janine (2021). Automation in DFT-based computational materials science. Trends Chem..

[4] Choudhary Kamal, DeCost Brian, Chen Chi, Jain Anubhav, Tavazza Francesca, Cohn Ryan, Park Cheol Woo, Choudhary Alok, Agrawal Ankit, Billinge Simon JL (2022). Recent advances and applications of deep learning methods in materials science. NPJ Computat. Mater..

[5] Goh Garrett B, Hodas Nathan O, Vishnu Abhinav (2017). Deep learning for computational chemistry. J. Comput. Chem..

[6] Korshunova Maria, Ginsburg Boris, Tropsha Alexander, Isayev Olexandr (2021). Openchem: A deep learning toolkit for computational chemistry and drug design. J. Chem. Inf. Model..

[7] Mater Adam C, Coote Michelle L (2019). Deep learning in chemistry. J. Chem. Inf. Model..

[8] Keith John A, Vassilev-Galindo Valentin, Cheng Bingqing, Chmiela Stefan, Gastegger Michael, Muller Klaus-Robert, Tkatchenko Alexandre (2021). Combining machine learning and computational chemistry for predictive insights into chemical systems. Chem. Rev..

[9] Goldman Brian B, Walters W Patrick (2006). Machine learning in computational chemistry. Ann. Rep. Comput. Chem..

[10] Marx Dominik, Hutter Jurg (2000). Ab initio molecular dynamics: Theory and implementation. Modern Methods Algorithms Quantum Chem..

[11] Tuckerman Mark E (2002). Ab initio molecular dynamics: basic concepts, current trends and novel applications. J. Phys.: Condens. Matter.

[12] Parr, R. G. Density functional theory. In *Electron Distributions and the Chemical Bond*, pp. 95–100. Springer, (1982).

[13] Cohen Aron J, Mori-Sánchez Paula, Yang Weitao (2012). Challenges for density functional theory. Chem. Rev..

[14] Verma Pragya, Truhlar Donald G (2020). Status and challenges of density functional theory. Trends Chem..

[15] Wang Xufei, Yuanda Xu, Zheng Han, Kuang Yu (2021). A scalable graph neural network method for developing an accurate force field of large flexible organic molecules. J. Phys. Chem. Lett..

[16] Smith Justin S, Isayev Olexandr, Roitberg Adrian E (2017). Ani-1: An extensible neural network potential with DFT accuracy at force field computational cost. Chem. Sci..

[17] Unke Oliver T, Meuwly Markus (2019). Physnet: A neural network for predicting energies, forces, dipole moments, and partial charges. J. Chem. Theory Comput..

[18] Coley Connor W, Jin Wengong, Rogers Luke, Jamison Timothy F, Jaakkola Tommi S, Green William H, Barzilay Regina, Jensen Klavs F (2019). A graph-convolutional neural network model for the prediction of chemical reactivity. Chem. Sci..

[19] Schütt Kristof T, Sauceda Huziel E, Kindermans P-J, Tkatchenko Alexandre, Müller K-R (2018). Schnet-a deep learning architecture for molecules and materials. J. Chem. Phys..

[20] Zhang Linfeng, Han Jiequn, Wang Han, Car Roberto, Weinan EJPRL (2018). Deep potential molecular dynamics: a scalable model with the accuracy of quantum mechanics. Phys. Rev. Lett..

[21] Ko Tsz Wai, Finkler Jonas A, Goedecker Stefan, Behler Jörg (2021). A fourth-generation high-dimensional neural network potential with accurate electrostatics including non-local charge transfer. Nat. Commun..

[22] Behler Jörg, Parrinello Michele (2007). Generalized neural-network representation of high-dimensional potential-energy surfaces. Phys. Rev. Lett..

[23] Ko Tsz Wai, Finkler Jonas A, Goedecker Stefan, Behler Jörg (2021). General-purpose machine learning potentials capturing nonlocal charge transfer. Acc. Chem. Res..

[24] Kresse Georg, Hafner Jürgen (1993). Ab initio molecular dynamics for liquid metals. Phys. Rev. B.

[25] Gastegger Michael, Behler Jörg, Marquetand Philipp (2017). Machine learning molecular dynamics for the simulation of infrared spectra. Chem. Sci..

[26] Jacobson Leif D, Stevenson James M, Ramezanghorbani Farhad, Ghoreishi Delaram, Leswing Karl, Harder Edward D, Abel Robert (2022). Transferable neural network potential energy surfaces for closed-shell organic molecules: Extension to ions. J. Chem. Theory Comput..

[27] Grimme Stefan, Ehrlich Stephan, Goerigk Lars (2011). Effect of the damping function in dispersion corrected density functional theory. J. Comput. Chem..

[28] Mardirossian Narbe, Head-Gordon Martin (2014). $$\omega$$b97x-v: A 10-parameter, range-separated hybrid, generalized gradient approximation density functional with nonlocal correlation, designed by a survival-of-the-fittest strategy. Phys. Chem. Chem. Phys..

[29] Dajnowicz S , Agarwal G, Stevenson JM, Jacobson LD, Ramezanghorbani F, Leswing K, Friesner RA, Halls MD, Abel R (2022). High-dimensional neural network potential for liquid electrolyte simulations. J. Phys. Chem. B.

[30] Ghasemi S Alireza, Hofstetter Albert, Saha Santanu, Goedecker Stefan (2015). Interatomic potentials for ionic systems with density functional accuracy based on charge densities obtained by a neural network. Phys. Rev. B.

[31] Gastegger Michael, Kauffmann Clemens, Behler Jörg, Marquetand Philipp (2016). Comparing the accuracy of high-dimensional neural network potentials and the systematic molecular fragmentation method: A benchmark study for all-trans alkanes. J. Chem. Phys..

[32] Unke, O. T., Stöhr, M., Ganscha, S., Unterthiner, T., Maennel, H., Kashubin, S., Ahlin, D., Gastegger, M., Sandonas, L. M., & Tkatchenko, A., *et al.* Accurate machine learned quantum-mechanical force fields for biomolecular simulations. arXiv preprint arXiv:2205.08306, (2022). 10.1126/sciadv.adn4397 PMC11809612 38579003

[33] Nagarajan R, Wang Chien-Chung (2000). Theory of surfactant aggregation in water/ethylene glycol mixed solvents. Langmuir.

[34] Hoogland FG, Boon JJ (2009). Development of MALDI-MS and nano-ESI-MS methodology for the full identification of poly (ethylene glycol) additives in artists’ acrylic paints. Int. J. Mass Spectrom..

[35] Hollis Jan M, Lovas Frank J, Jewell Philip R, Coudert LH (2002). Interstellar antifreeze: Ethylene glycol. Astrophys. J..

[36] Boatman Rodney J, Knaak James B (2001). Ethers of Ethylene Glycol and Derivatives.

[37] Jung-Tang Wu, Hsu Steve Lien-Chung, Tsai Ming-Hsiu, Hwang Weng-Sing (2009). Conductive silver patterns via ethylene glycol vapor reduction of ink-jet printed silver nitrate tracks on a polyimide substrate. Thin Solid Films.

[38] Chen Guo-Qiang, Patel Martin K (2012). Plastics derived from biological sources: Present and future: A technical and environmental review. Chem. Rev..

[39] Sharma Sadhana, Johnson Robert W, Desai Tejal A (2004). Evaluation of the stability of nonfouling ultrathin poly (ethylene glycol) films for silicon-based microdevices. Langmuir.

[40] Cheng Yu-Wei, Lin Yen-Ting, Liu Kun-Ho, Chen Jung-San, Wang Shih-Hsuan, Liu Ting-Yu (2022). In situ and initiator-free atmospheric plasma-induced functionalization of poly (ethylene glycol) methacrylate on nonwoven cosmetic masks for the evaluation of the bacteria inhibitory effect. Colloids Surf. A.

[41] Zafarani-Moattar Mohammed Taghi, Shekaari Hemayat, Haji Agha Elnaz Mazaher (2019). Phase equilibrium study in aqueous solutions containing ionic liquid 1-butyl-3-methyl imidazolium chloride and poly (propylene glycol) 400 or poly (ethylene glycol) dimethyl ether 250 via a vapor–liquid equilibrium study at t= 298.15 k.. J. Chem. Eng. Data.

[42] Ijardar Sushma P (2020). Deep eutectic solvents composed of tetrabutylammonium bromide and peg: Density, speed of sound and viscosity as a function of temperature. J. Chem. Thermodyn..

[43] DuBay Kateri H, Hall Michelle Lynn, Hughes Thomas F, Chuanjie Wu, Reichman David R, Friesner Richard A (2012). Accurate force field development for modeling conjugated polymers. J. Chem. Theory Comput..

[44] Fang Chan-En, Tsai Yi-Chen, Scheurer Christoph, Chiu Chi-Cheng (2021). Revised atomic charges for OPLS force field model of poly (ethylene oxide): Benchmarks and applications in polymer electrolyte. Polymers.

[45] Heinz Hendrik, Lin Tzu-Jen, Kishore Mishra Ratan, Emami Fateme S (2013). Thermodynamically consistent force fields for the assembly of inorganic, organic, and biological nanostructures: the interface force field. Langmuir.

[46] Szefczyk Borys, DS Cordeiro M Natalia (2011). Physical properties at the base for the development of an all-atom force field for ethylene glycol. J. Phys. Chem. B.

[47] Saiz L, Padro JA, Guardia E (2001). Structure of liquid ethylene glycol: A molecular dynamics simulation study with different force fields. J. Chem. Phys..

[48] Caleman Carl, Van Maaren Paul J, Hong Minyan, Hub Jochen S, Costa Luciano T, Van Der Spoel David (2012). Force field benchmark of organic liquids: density, enthalpy of vaporization, heat capacities, surface tension, isothermal compressibility, volumetric expansion coefficient, and dielectric constant. J. Chem. Theory Comput..

[49] Haynes William M, Lide David R, Bruno Thomas J (2016). CRC Handbook of Chemistry and Physics.

[50] Siu Shirley WI, Pluhackova Kristyna, Bockmann Rainer A (2012). Optimization of the OPLS-aa force field for long hydrocarbons. J. Chem. Theory Comput..

[51] Chao Lu, Chuanjie Wu, Ghoreishi Delaram, Chen Wei, Wang Lingle, Damm Wolfgang, Ross Gregory A, Dahlgren Markus K, Russell Ellery, Von Bargen Christopher D (2021). Opls 4: Improving force field accuracy on challenging regimes of chemical space. J. Chem. Theory Comput..

[52] Materials Science Suite, verion 2022-2. Schrodinger, LLC: New York. URL https://www.schrodinger.com/platform/materials-science.

[53] Afzal, M. A. F., Sanders, J. M., Goldberg, A., Browning, A. R. & Halls M. D. Using molecular simulation with high-temperature composites resins. (2019a).

[54] Mohanty, S., Stevenson, J., Browning, A. R., Jacobson, L. D., Leswing, K., Halls, M. D. & Faiz, A. M. A. Development of Scalable and Generalizable Machine Learned Force Field for Polymers. 12 (2022). 10.6084/m9.figshare.21720881.v1. URL https://figshare.com/articles/dataset/Development_of_Scalable_and_Generalizable_Machine_Learned_Force_Field_for_Polymers/21720881. 10.1038/s41598-023-43804-5 PMC10567837 37821501

[55] Weigend Florian (2006). Accurate coulomb-fitting basis sets for H to Rn. Phys. Chem. Chem. Phys..

[56] Turney Justin M, Simmonett Andrew C, Parrish Robert M, Hohenstein Edward G, Evangelista FrancescoA, Fermann JustinT, Mintz BenjaminJ, Burns Lori A, Wilke Jeremiah J, Abrams Micah L (2012). Psi4: An open-source ab initio electronic structure program. Wiley Interdisciplinary Rev. Comput. Mol. Sci..

[57] Goerigk Lars, Hansen Andreas, Bauer Christoph, Ehrlich Stephan, Najibi Asim, Grimme Stefan (2017). A look at the density functional theory zoo with the advanced gmtkn55 database for general main group thermochemistry, kinetics and noncovalent interactions. Phys. Chem. Chem. Phys..

[58] Grimme Stefan (2006). Semiempirical GGA-type density functional constructed with a long-range dispersion correction. J. Comput. Chem..

[59] Chai Jeng-Da, Head-Gordon Martin (2008). Long-range corrected hybrid density functionals with damped atom-atom dispersion corrections. Phys. Chem. Chem. Phys..

[60] Cipolla, R., Gal, Y., & Kendall, A. Multi-task learning using uncertainty to weigh losses for scene geometry and semantics. *2018 IEEE/CVF Conference on Computer Vision and Pattern Recognition*, pp. 7482–7491, (2018).

[61] Bannwarth Christoph, Ehlert Sebastian, Grimme Stefan (2019). GFN2-xTB—an accurate and broadly parametrized self-consistent tight-binding quantum chemical method with multipole electrostatics and density-dependent dispersion contributions. J. Chem. Theory Comput..

[62] Hoffmann Markus M, Horowitz Rachel H, Gutmann Torsten, Buntkowsky Gerd (2021). Densities, viscosities, and self-diffusion coefficients of ethylene glycol oligomers. J. Chem. Eng. Data.

[63] Afzal Mohammad Atif Faiz, Browning Andrea R, Goldberg Alexander, Halls Mathew D, Gavartin Jacob L, Morisato Tsuguo, Hughes Thomas F, Giesen David J, Goose Joseph E (2020). High-throughput molecular dynamics simulations and validation of thermophysical properties of polymers for various applications. ACS Appl. Polym. Mater..

[64] Afzal MAF, Sonpal A, Haghighatlari M, Schultz AJ, Hachmann J (2019). A deep neural network model for packing density predictions and its application in the study of 1.5 million organic molecules. Chem. Sci..

[65] Berens Peter H, Mackay Donald HJ, White Gary M, Wilson Kent R (1983). Thermodynamics and quantum corrections from molecular dynamics for liquid water. J. Chem. Phys..

[66] Trivedi S, Bhanot C, Pandey S (2010). Densities of poly (ethylene glycol)+ water over the temperature range (283.15 to 363.15) k. J. Chem. Thermodyn..

[67] Stevenson, J. M., Jacobson, L. D., Zhao, Y., Wu, C., Maple, J., Leswing, K., Harder, E., & Abel, R. Schrodinger-ANI: An eight-element neural network interaction potential with greatly expanded coverage of druglike chemical space. arXiv preprint arXiv:1912.05079, (2019).

