# Supplementary material for: Development of scalable and generalizable machine learned force field for polymers
Source: Sci Rep. 2023 Oct 11;13:17251. doi: 10.1038/s41598-023-43804-5 (PMC10567837; doi:10.1038/s41598-023-43804-5)
Supplement: Supplementary file 1 — Supplementary Information. [file 41598_2023_43804_MOESM1_ESM.pdf]

Supplementary Information:
Development of Scalable and Generalizable Machine Learned Force Field for Polymers

Shaswat Mohanty[a], James Stevenson[c], Andrea R. Browning[b], Leif Jacobson[b], Karl Leswing[c], Mathew D. Halls[d], Mohammad Atif Faiz Afzal[b]

[a]*Department of Mechanical Engineering, Stanford University, California 94305-4040, USA*
[b]*Schrödinger, Inc., Portland, Oregon 97204, United States*
[c]*Schrödinger, Inc., New York 10036, United States*
[d]*Schrödinger, Inc., San Diego, California 92121, United States*

In this document, we elucidate a few of the methods and the results that have been truncated for brevity in the main text.

S1. Methods

In this particular section, we discuss the quantitative specifics and details of the various methods employed in generating the training dataset for the QRNN model training and inference.

S1.1. Sample preparation

The ten-step relaxation carried out on the initialized configurations is given by:

- Brownian Dynamics on the initialized configuration for 100 ps at 10 K with a timestep of 1 fs.
- NVT simulation at 300 K for 50 ps with a timestep of 1 fs.
- NVT simulation at 70 K for 200 ps with a timestep of 1 fs.
- NPT simulation with an external pressure of 1 atm at 300K for 50 ps with a timestep 1 fs.
- NPT simulation with an external pressure of 1 atm at 300K for 200 ps with a timestep of 2 fs.
- NPT simulation with an external pressure of 100 atm at 300K for 10 ns with a timestep 2 fs.
- NPT simulation with an external pressure of 1 atm at 300K for 10 ns with a timestep of 2 fs.

- The cell size is averaged over the last 2 ns of the previous stage for the volume of the NVT simulation in the next stage.
- NVT simulation at 300 K for 5 ns with a timestep of 2 fs.

The relaxed configuration at the end of this is used as the starting configuration for QRNN MD.

S1.2. Dataset preparation for QRNN training

The sampled conformational clusters also include density variations in addition to the bond, angle, and dihedral deformations. On the other hand, decomposed samples only include bond breakage (i.e. extreme bond sampling). The breakdown of the regularly identified clusters and the subsequent number of conformational and decomposed clusters for the different N-molecule clusters for the monomer, dimer, and trimer of ethylene glycol are given in Table S1.

Ethylene Glycol Clusters

N-molecule	N_{atoms}	Clusters	Sampled Clusters	Decomposed Clusters
1	10	793	5000	20000
2	20	2931	20000	10000
3	30	868	20000	5000
4	40	366	25000	4000
5	50	187	15000	2000
6	60	108	10000	-
7	70	68	6000	-
8	80	45	2000	-

Diethylene Glycol Clusters

N-molecule	N_{atoms}	Clusters	Sampled Clusters	Decomposed Clusters
1	17	480	20000	20000
2	34	2157	20000	10000
3	51	639	15000	5000
4	68	269	7500	-
5	85	138	2000	-
6	102	79	1000	-

Triethylene Glycol Clusters

N-molecule	N_{atoms}	Clusters	Sampled Clusters	Decomposed Clusters
1	24	333	20000	20000
2	48	1607	15000	10000
3	72	476	6000	-
4	96	200	1000	-

Table S1: Number of original clusters extracted from OPLS4 MD, conformational sampled clusters, and decomposed sampled clusters for ethylene glycol, diethylene glycol, and triethylene glycol.

S1.3. Active learning

We generated additional 800,000 clusters using the split between the different N-molecule clusters of the monomer, dimer, and trimer of ethylene glycol, as shown in Table S2.

Ethylene Glycol Clusters

N-molecule	N_{atoms}	Clusters	Sampled Clusters
1	10	300	80000
2	20	4266	160000
3	30	1283	140000
4	40	533	30000
5	50	272	15000
6	60	157	5000
7	70	98	2000

Diethylene Glycol Clusters

N-molecule	N_{atoms}	Clusters	Sampled Clusters
1	17	178	150000
2	34	2468	80000
3	51	730	15000
4	68	308	5000
5	85	156	2000

Triethylene Glycol Clusters

N-molecule	N_{atoms}	Clusters	Sampled Clusters
1	24	126	120000
2	48	1680	15000
3	72	493	2000

Table S2: Number of clusters extracted from QRNN MD and corresponding conformational sampled clusters for ethylene glycol, diethylene glycol, and triethylene glycol that are used for filtering for the first round of active learning.

For the second round of active learning, we carry out the same analysis only from the extracted molecular clusters from our QRNN simulations, as shown in Table S3. We do not carry out any conformational sampling at this stage.

Ethylene Glycol Clusters

N-molecule	N_{atoms}	Extracted Clusters
1	10	7500
2	20	37180
3	30	10991
4	40	4625
5	50	2356

Diethylene Glycol Clusters

N-molecule	N_{atoms}	Extracted Clusters
1	17	22250
2	34	116548
3	51	34405

Triethylene Glycol Clusters

N-molecule	N_{atoms}	Extracted Clusters
1	24	15750
2	48	61566

Table S3: Number of extracted clusters for ethylene glycol, diethylene glycol, and triethylene glycol that are used for filtering for the second round of active learning.

S2. Results

The model performance is in good agreement with the reference results with $R^2 \sim 1$, as shown in Fig. S1, with an RMSE of 1.84×10^{-3} eV/atom for energy, RMSE of 7.80×10^{-3} for charges, and RMSE of 3.08×10^{-1} eV/Å for forces .

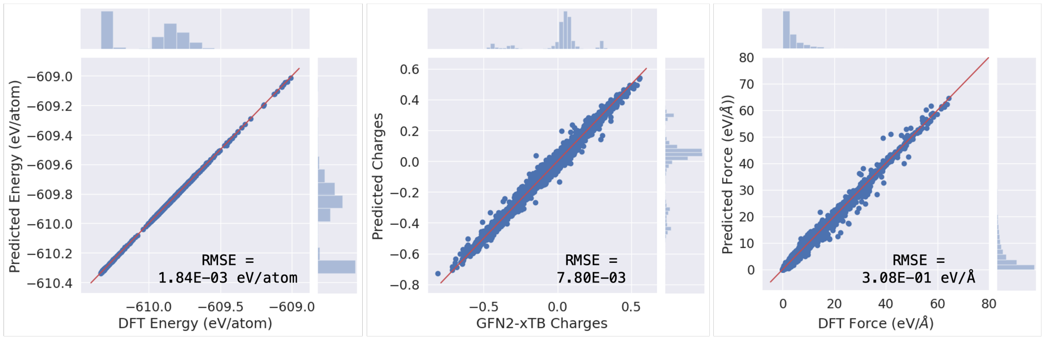

Figure S1: Parity plot showing the model prediction of energies, charges, and forces against the reference values for 1486 72-atom configurations.

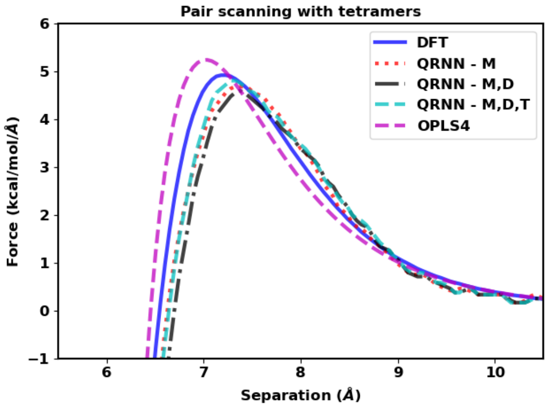

Figure S2: Calculated force to separate a pair of tetramer molecules from a given separation distance. The force is calculated by taking the gradient of the energy curves shown in Fig. 1 of the main text.

The tabulated results from the density, specific heat capacity and self-diffusivity obtained from the `QRNN - M`, `QRNN - M,D`, and `QRNN - M,D,T` are given in this section.

N-mer	Density (g/cm^3)				
	Experimental	OPLS4 MD	`QRNN - M`	`QRNN - M,D`	`QRNN - M,D,T`
1-mer	1.1108 [1]	1.082 ± 0.002	1.036 ± 0.001	1.015 ± 0.001	1.058 ± 0.001
2-mer	1.1124 [2]	1.095 ± 0.003	1.045 ± 0.002	1.039 ± 0.002	1.068 ± 0.002
3-mer	1.1192 [2]	1.105 ± 0.006	1.074 ± 0.001	1.053 ± 0.003	1.074 ± 0.004
4-mer	1.1185 [2]	1.113 ± 0.009	1.073 ± 0.002	1.059 ± 0.002	1.073 ± 0.001
5-mer	1.1201 [2]	1.115 ± 0.005	1.073 ± 0.002	1.063 ± 0.003	1.073 ± 0.002
6-mer	1.1227 [2]	1.117 ± 0.009	1.071 ± 0.002	1.065 ± 0.003	1.061 ± 0.002
7-mer	1.122 [2]	1.121 ± 0.007	1.069 ± 0.002	1.065 ± 0.004	1.069 ± 0.002
8-mer	1.12 [2]	1.121 ± 0.005	1.069 ± 0.002	1.063 ± 0.003	1.069 ± 0.002
9-mer	1.1221 [2]	1.124 ± 0.006	1.070 ± 0.002	1.067 ± 0.005	1.070 ± 0.002
10-mer	-	1.121 ± 0.004	1.067 ± 0.002	1.067 ± 0.003	1.069 ± 0.002

Table S4: Comparison of the experimental density of ethylene glycol oligomers with the classically obtained density from MD simulations using an OPLS4 force field and with the machine-learned force field simulations.

N-mer	D (μm^2/s)				
	Experimental	OPLS4 MD	`QRNN - M`	`QRNN - M,D`	`QRNN - M,D,T`
1-mer	88.2 [3]	125.91 ± 5.80	199.34 ± 21.03	422.57 ± 67.07	135.28 ± 16.24
2-mer	61.7 [2]	18.67 ± 1.86	71.08 ± 11.88	142.23 ± 15.62	34.87 ± 8.36
3-mer	40.57 [2]	4.93 ± 0.58	66.61 ± 20.39	90.97 ± 15.64	41.84 ± 20.39
4-mer	33.59 [2]	2.18 ± 0.31	51.86 ± 18.38	56.69 ± 12.68	46.74 ± 18.38
5-mer	25.83 [2]	1.57 ± 0.58	46.59 ± 11.16	45.19 ± 6.16	33.06 ± 11.16
6-mer	21.22 [2]	0.92 ± 0.25	45.14 ± 10.65	34.35 ± 14.35	29.38 ± 10.65
7-mer	17.48 [2]	0.73 ± 0.23	37.39 ± 9.74	28.02 ± 9.31	31.13 ± 9.74
8-mer	14.68 [2]	0.58 ± 0.13	37.94 ± 12.70	24.07 ± 4.43	29.53 ± 12.71
9-mer	12.11 [2]	0.53 ± 0.11	29.79 ± 11.67	18.94 ± 4.62	20.91 ± 11.68
10-mer	-	0.54 ± 0.13	. 32.40 ± 18.39	18.16 ± 7.12	21.19 ± 6.52

Table S5: Comparison of the experimental self-diffusivity of ethylene glycol oligomers with the classically obtained density from MD simulations using an OPLS4 force field and with the machine-learned force field simulations.

N-mer	c_p (J/g-K)				
	Experimental	OPLS4 MD	`QRNN - M`	`QRNN - M,D`	`QRNN - M,D,T`
1-mer	2.422 [4]	4.54 ± 0.23	3.25 ± 0.07	3.11 ± 0.10	3.36 ± 0.12
2-mer	2.298 [5]	4.41 ± 0.29	3.15 ± 0.21	3.08 ± 0.16	3.54 ± 0.53
3-mer	2.22 [5]	4.04 ± 0.23	2.87 ± 0.12	2.93 ± 0.14	3.08 ± 0.24
4-mer	2.20 [5]	4.16 ± 0.45	2.85 ± 0.20	2.97 ± 0.14	3.03 ± 0.16
5-mer	2.172 [6]	4.27 ± 0.56	2.82 ± 0.13	2.93 ± 0.22	2.89 ± 0.11
6-mer	2.196 [5]	4.05 ± 0.29	2.85 ± 0.16	2.87 ± 0.12	3.08 ± 0.18
7-mer	-	3.61 ± 0.24	2.89 ± 0.13	2.92 ± 0.30	2.79 ± 0.11
8-mer	-	3.83 ± 0.19	2.80 ± 0.12	2.99 ± 0.29	2.92 ± 0.19
9-mer	-	3.89 ± 0.36	2.80 ± 0.14	3.02 ± 0.33	2.88 ± 0.12
10-mer	-	3.86 ± 0.28	2.78 ± 0.12	2.95 ± 0.16	2.82 ± 0.17

Table S6: Comparison of the experimental specific heat capacity of ethylene glycol oligomers with the classically obtained density from MD simulations using an OPLS4 force field and with the machine-learned force field simulations.

The collated experimental values of the properties we are interested in can be found in Table S7.

Molecule	ρ (g/cm^3)	c_p (J/g-K)	D (μm^2/s)
1-mer	1.1108 [1]	2.422 [4]	88.2 [3]
2-mer	1.1124 [2]	2.298 [5]	61.7 [2]
3-mer	1.1192 [2]	2.22 [5]	40.57 [2]
4-mer	1.1185 [2]	2.20 [5]	33.59 [2]
5-mer	1.1201 [2]	2.172 [6]	25.83 [2]
6-mer	1.1227 [2]	2.196 [5]	21.22 [2]
7-mer	1.122 [2]	-	17.48 [2]
8-mer	1.12 [2]	-	14.68 [2]
9-mer	1.1221 [2]	-	12.11 [2]
10-mer	1.1	-	-

Table S7: Ethylene glycol oligomer properties: density (ρ), viscosity (η), specific heat (c_p) and self-diffusivity (D) at 300K (NIST WEbBook)

In addition, we also look at the figures of predicted density, specific heat capacity, and self-diffusivity along with how well they correlate with the experimentally obtained values. A high correlation even with an inaccurate prediction can prove fruitful since the experimental values can be obtained by applying a linear correction factor to the MD-predicted properties.

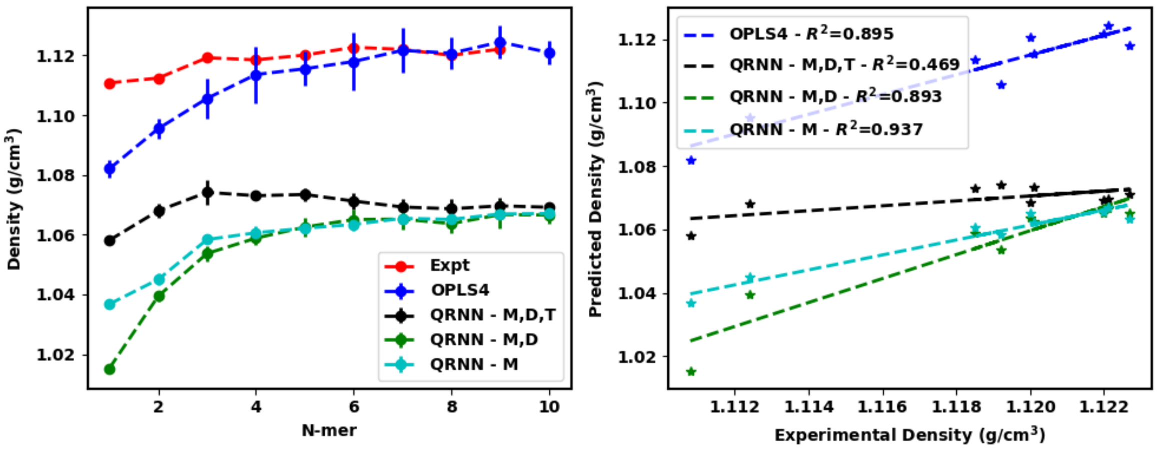

Figure S3: (a) Predicted density against the experimental density and the (b) correlation between them.

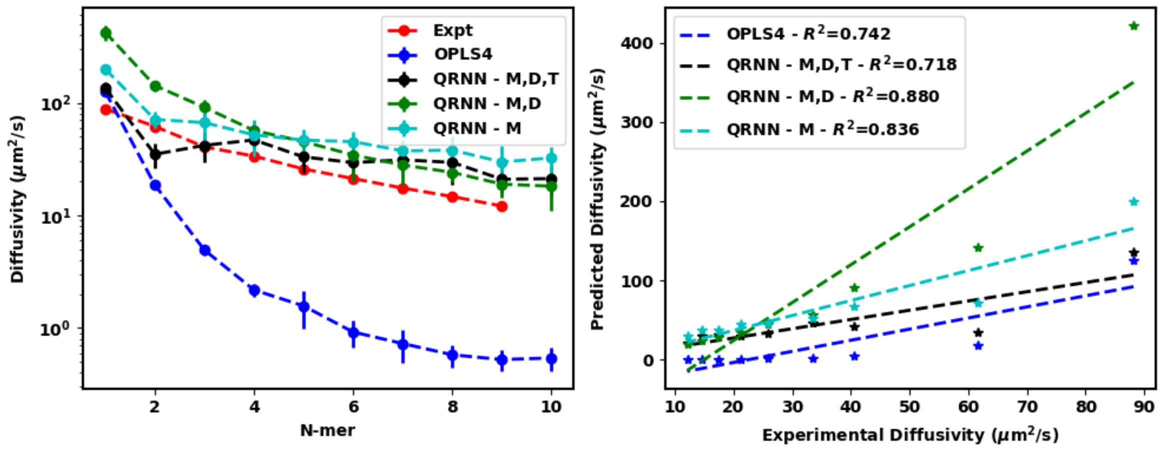

Figure S4: (a) Predicted diffusivity against the experimental diffusivity and the (b) correlation between them.

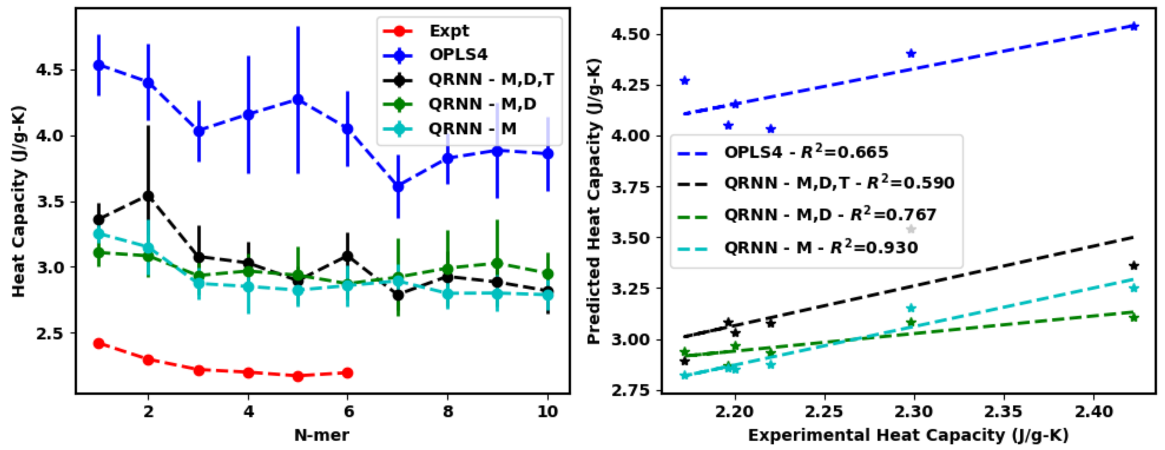

Figure S5: (a) Predicted specific heat capacity against the experimental specific heat capacity and the (b) correlation between them.

We see that the first three models of `QRNN - M`, `QRNN - M,D`, and `QRNN - M,D,T` do have their benefits in the prediction of self-diffusivity, however, they are not well correlated with the experimental values of the properties that we are exploring. However, the models generated after the first round of active learning, show not just more accurate predictions in the experimental properties but they also show a better correlation with the experimental values. In total, we carry out two rounds of active learning with combinations of either training a whole new model or transfer learning from the previously learned model. Here we present the results from the most promising set of models trained from the two rounds of active learning.

S2.1. First round of active learning

In this section, we look at the performance of two models trained after adding the new samples from the active learning analysis. We compare both these models against `QRNN - M,D,T` which is one of the initially trained models. The two models we compare are: (i) `QRNN - AL-TL` which is transfer learns on `QRNN - M,D,T` and uses the composite dataset of the old samples and the new active learning ones; (ii) `QRNN - AL-TL-xTB` which uses transfer learns in a similar way as `QRNN - AL-TL` but instead of using the dipole moments as a feature for learning, we use the extended tight binding (xTB) charges.

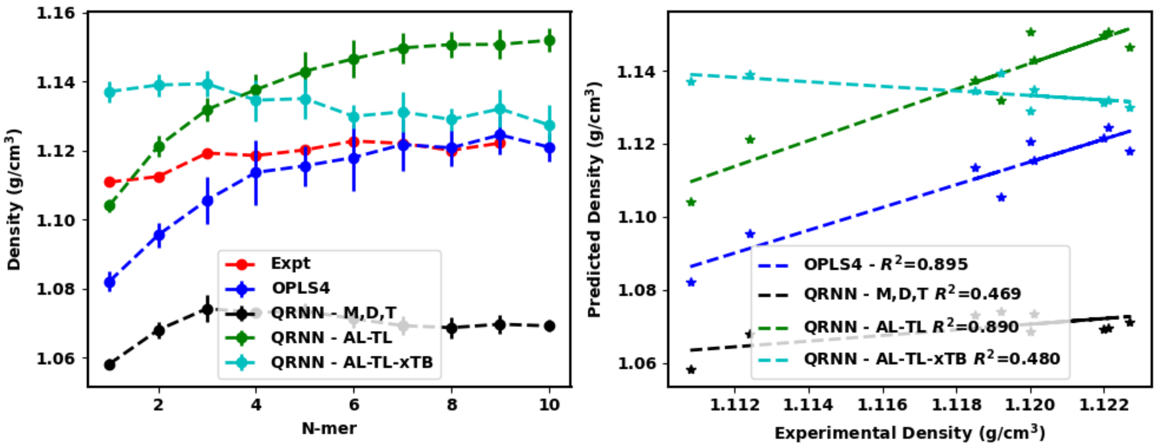

Figure S6: (a) Predicted density against the experimental density and the (b) correlation between them.

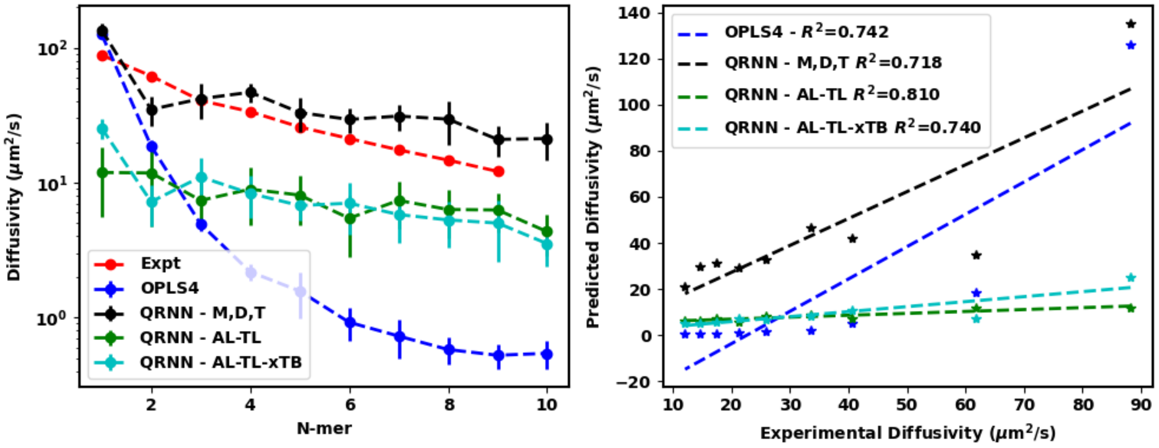

Figure S7: (a) Predicted diffusivity against the experimental diffusivity and the (b) correlation between them.

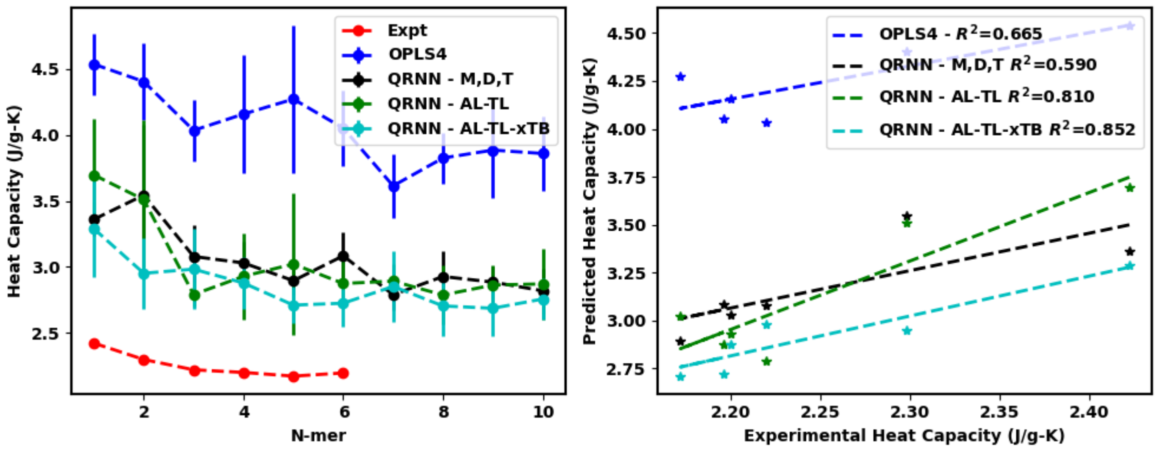

Figure S8: (a) Predicted specific heat capacity against the experimental specific heat capacity and the (b) correlation between them.

These newer models are not just qualitatively closer to predicting the experimental values but they also allow us to come up with a linear correction relation to be able to estimate the experimental density, diffusivity or specific heat capacity directly from the QRNN MD predictions.

The dimer scanning results from the first round of active learning which are not shown in the manuscript are given below.

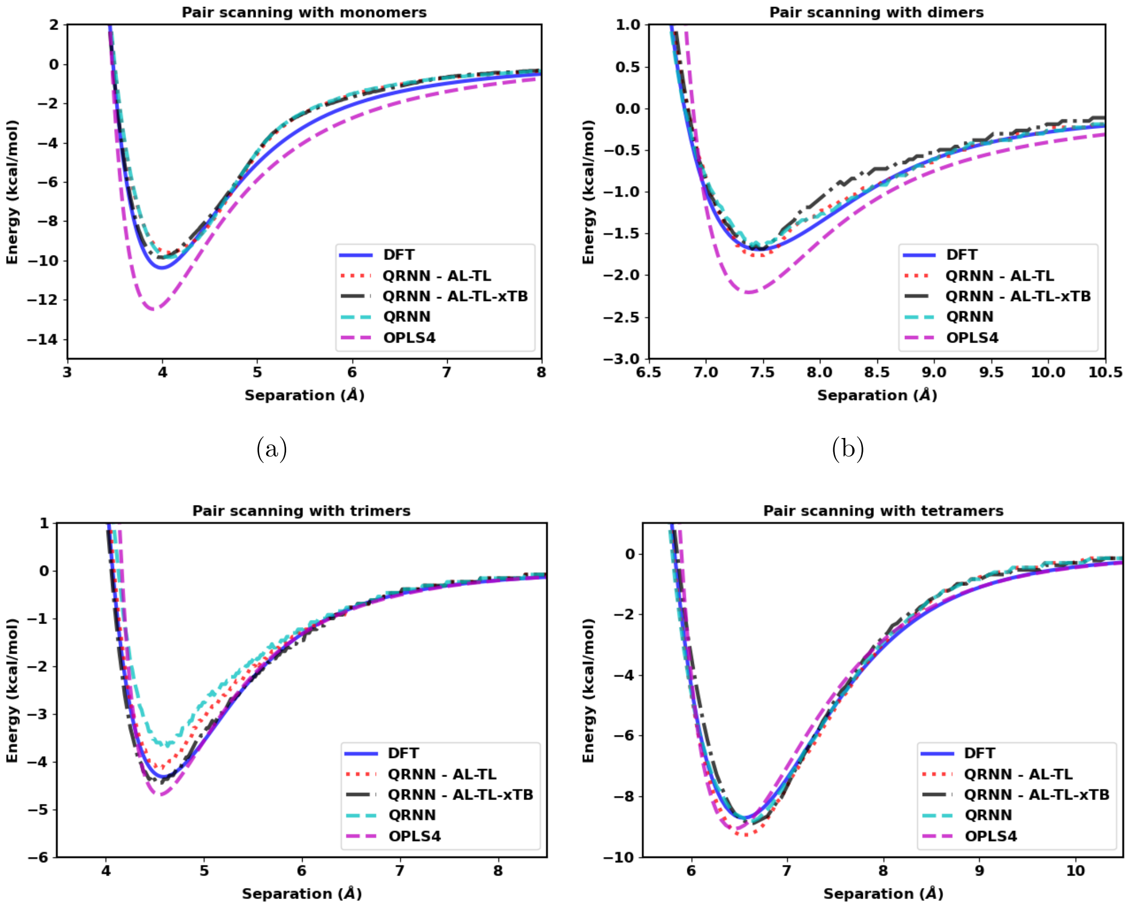

(a) (b) (c) (d)

Figure S9: Separation energy between two molecules as a function of separation distance compared between the DFT calculation, OPLS4 force field, and the machine-learned force field for the (a) monomers, (b) dimers, (c) trimers and (d) tetramers of ethylene glycol.

S2.2. Second round of active learning

As established from the first round of learning, training on xTB charges can turn out to be beneficial. As a result, we carry out a second round active-learning and add these new samples to our training dataset. We then train a number of models that either use the dipole moments or xTB charges along with the forces and energy as the learning features. The best model `QRNN - AL2.0-TL-xTB` is compared against `QRNN - M,D,T` and `QRNN - AL-TL` from the previous rounds to show the superior performance.

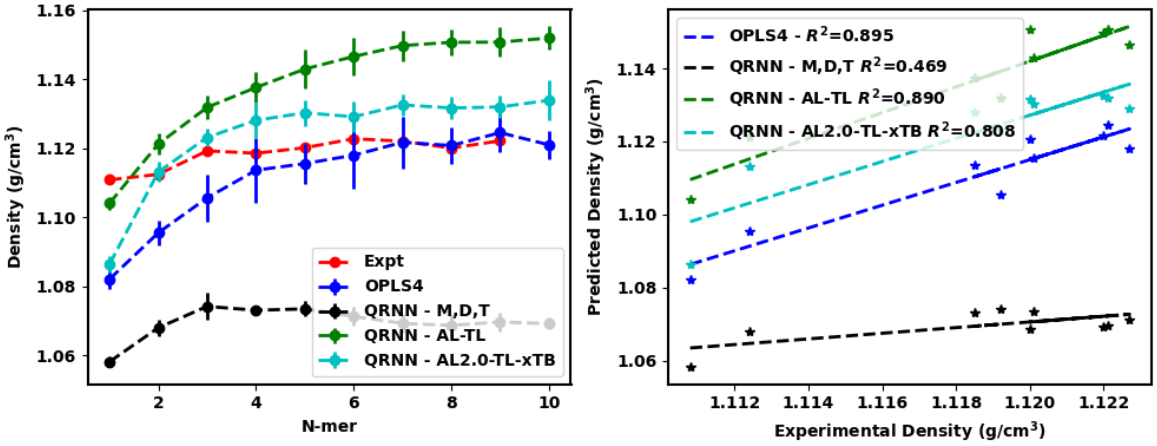

Figure S10: (a) Predicted density against the experimental density and the (b) correlation between them.

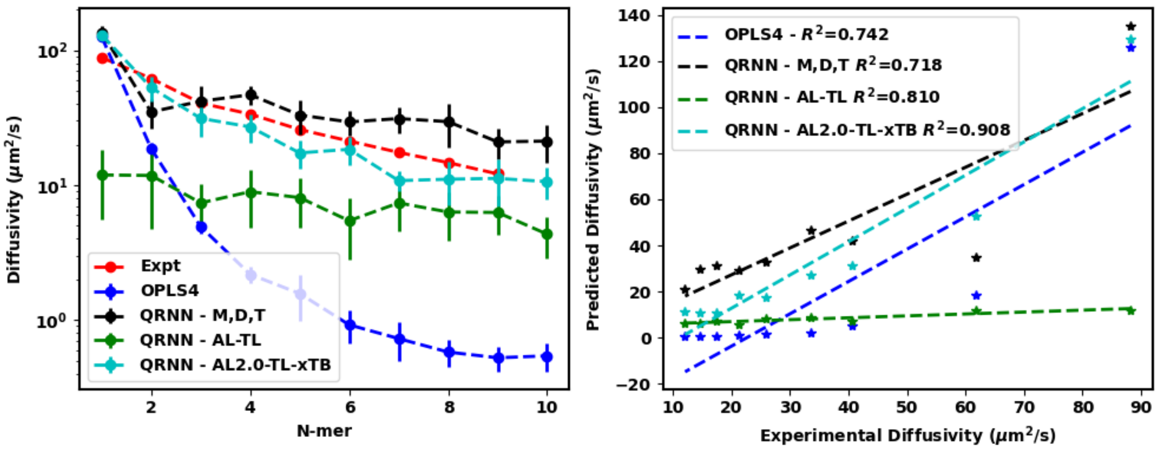

Figure S11: (a) Predicted diffusivity against the experimental diffusivity and the (b) correlation between them.

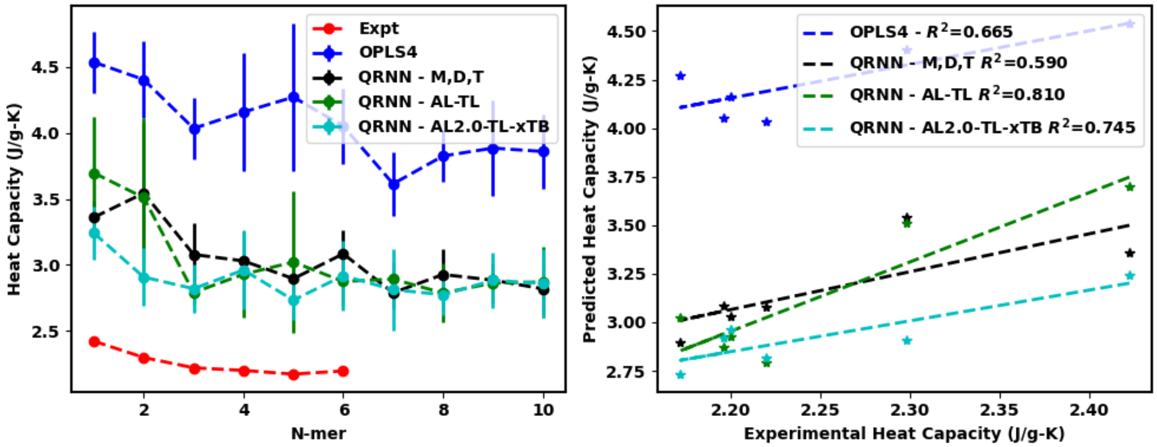

Figure S12: (a) Predicted specific heat capacity against the experimental specific heat capacity and the (b) correlation between them.

A change from training on xTB atomic partial charge labels instead of DFT dipole moments and an incremental change in the model performance with each cycle of active learning can be seen in Fig. S13. Our findings indicate that the xTB charge model does better than the DFT dipole moment model. Additionally, we have observed gradual enhancements in the results through consecutive active learning cycles within the xTB charge model. Therefore, it is the combined utilization of xTB charges and active learning that leads to the most favorable outcomes.

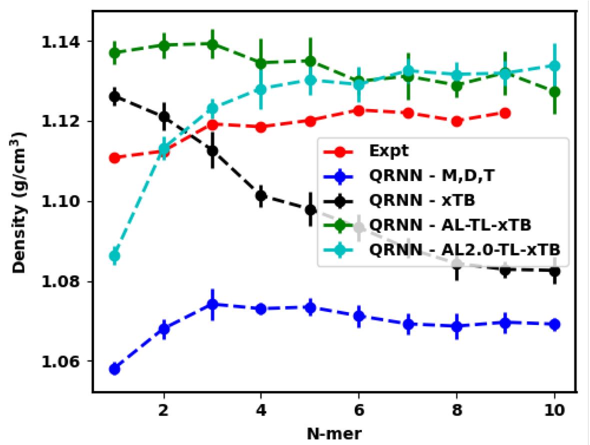

Figure S13: Comparison of the xTB charge model (QRNN - xTB) with DFT dipole moment model (QRNN - M,D,T)) and incremental improvements to the xTB charge model with active learning (QRNN - AL-TL-xTB, QRNN - AL2.0-TL-xTB).

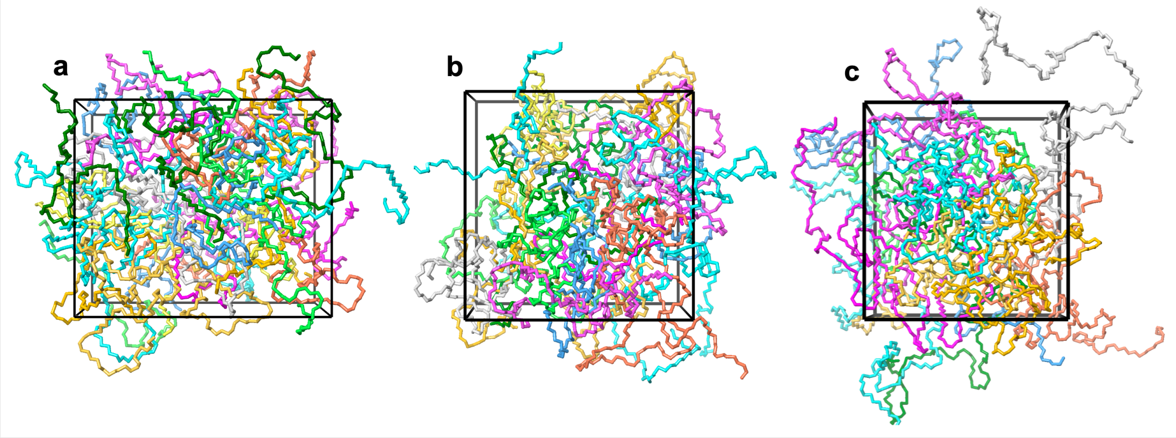

Figure S14: Equilibrated simulation cell containing (a) 25-mer, (b) 50-mer and (c) 100-mer chains of PEG.

References

[1] Engineering Toolbox. Ethylene - Density and Specific Weight vs. Temperature and Pressure. https://www.engineeringtoolbox.com/ethylene-ethene-acetene-C2H4-density-specific-weight-temperature-pressure-d_2105.html, 2018. [Online; accessed 19-July-2022].

[2] Markus M Hoffmann, Rachel H Horowitz, Torsten Gutmann, and Gerd Buntkowsky. Densities, viscosities, and self-diffusion coefficients of ethylene glycol oligomers. *Journal of Chemical & Engineering Data*, 66(6):2480–2500, 2021.

[3] William M Spees, Sheng-Kwei Song, Joel R Garbow, Jeffrey J Neil, and Joseph JH Ackerman. Use of ethylene glycol to evaluate gradient performance in gradient-intensive diffusion mr sequences. *Magnetic resonance in medicine*, 68(1):319–324, 2012.

[4] PN Nikolaev and IB Rabinovich. Heat capacity of ethylene glycol and ethylene deuteroglycol in the temperature range 80–300k. *Zhur. Fiz. Khim*, 41:2191–2194, 1967.

[5] ZI Zaripov. Experimental study of the isobaric heat capacity of liquid organic compounds with molecular weights of up to 4000. *Teplomassoobmen Teplofiz*, 1982.

[6] Meta A Stephens and William S Tamplin. Saturated liquid specific heats of ethylene glycol homologs. *Journal of Chemical and Engineering Data*, 24(2):81–82, 1979.
